# Monocytes Regulate the Mechanism of T-cell Death by Inducing Fas-Mediated Apoptosis during Bacterial Infection

**DOI:** 10.1371/journal.ppat.1002814

**Published:** 2012-07-19

**Authors:** Marc Daigneault, Thushan I. De Silva, Martin A. Bewley, Julie A. Preston, Helen M. Marriott, Andrea M. Mitchell, Timothy J. Mitchell, Robert C. Read, Moira K. B. Whyte, David H. Dockrell

**Affiliations:** 1 Department of Infection and Immunity, University of Sheffield Medical School, Sheffield, United Kingdom; 2 Sheffield Teaching Hospitals, Sheffield, United Kingdom; 3 Institute of Microbiology and Infection, School of Immunity and Infection, University of Birmingham, Birmingham, United Kingdom; National Institute of Allergy and Infectious Diseases, National Institutes of Health, United States of America

## Abstract

Monocytes and T-cells are critical to the host response to acute bacterial infection but monocytes are primarily viewed as amplifying the inflammatory signal. The mechanisms of cell death regulating T-cell numbers at sites of infection are incompletely characterized. T-cell death in cultures of peripheral blood mononuclear cells (PBMC) showed ‘classic’ features of apoptosis following exposure to pneumococci. Conversely, purified CD3^+^ T-cells cultured with pneumococci demonstrated necrosis with membrane permeabilization. The death of purified CD3^+^ T-cells was not inhibited by necrostatin, but required the bacterial toxin pneumolysin. Apoptosis of CD3^+^ T-cells in PBMC cultures required ‘classical’ CD14^+^ monocytes, which enhanced T-cell activation. CD3^+^ T-cell death was enhanced in HIV-seropositive individuals. Monocyte-mediated CD3^+^ T-cell apoptotic death was Fas-dependent both *in vitro* and *in vivo*. In the early stages of the T-cell dependent host response to pneumococci reduced Fas ligand mediated T-cell apoptosis was associated with decreased bacterial clearance in the lung and increased bacteremia. In summary monocytes converted pathogen-associated necrosis into Fas-dependent apoptosis and regulated levels of activated T-cells at sites of acute bacterial infection. These changes were associated with enhanced bacterial clearance in the lung and reduced levels of invasive pneumococcal disease.

## Author Summary

T-cells are important contributors to the early host response to pneumonia, but their numbers must be tightly regulated to limit inflammatory lung injury. Cell death regulates T-cell numbers but the mechanism of execution influences the inflammatory cost with apoptosis viewed as predominantly anti-inflammatory and necrosis as pro-inflammatory. We show that monocytes determine the mechanism of T-cell death during acute bacterial infection. Monocytes triggered Fas-dependent T-cell apoptosis but in the absence of monocytes T-cells died by necrosis, which required the pneumococcal virulence factor pneumolysin. We also show that Fas ligand is required to regulate the early T-cell dependent host response to pneumococci during pneumonia. Although monocytes have previously been associated with enhancement of the inflammatory response our results imply that a key role of monocytes is to dampen the inflammatory response through induction of Fas-mediated apoptosis of activated T-cells during *S. pneumoniae* pneumonia. Our data identifies a critical and unrecognized regulatory role for monocytes during pneumonia.

## Introduction

Innate immunity is critical for the rapid recognition and response to pathogenic micro-organisms [1]. A complex relationship exists between innate immune responses and T-cells. Innate immune responses recruit and activate T-cells at sites of infection but T-cells in turn regulate phagocyte function and can therefore modify inflammatory responses. Monocytes are key effectors of the innate immune response to bacteria and contribute to recruitment of T-cells at sites of infection [2]. In contrast to differentiated macrophages, however, monocytes have not been viewed as having a major role in the downregulation of the inflammatory response [3].

*Streptococcus pneumoniae* is one of the leading causes of infection-related mortality globally [4]. T-cells are key to host defense against pneumococci, making this a useful model with which to study the regulation of T-cells during bacterial infection [5], [6]. CD4^+^ T-cells are found at sites of pneumococcal colonization in the upper airway [7] and T-cells migrate to sites of infection in the lung [8]. In murine models CD4^+^ T-cell Th17 responses facilitate clearance of colonizing bacteria [7], [9] while CD4^+^ T-cells enhance clearance of bacteria from the lungs [5]. Other studies have emphasised an important role for CD8^+^ T-cells during pneumococcal pneumonia by demonstrating CD8^+^ T-cells limit the extent of the inflammatory response [10]. Despite these observations, CD4^+^ T-cell inhibition may also limit inappropriate degrees of inflammation in some models of pneumococcal infection and improve disease outcome, emphasizing that numbers of T-cell populations must be carefully regulated to ensure effective clearance of bacteria while limiting lung pathology [10],[11].

There is limited information on how T-cell numbers are regulated during the immune response to pneumococci and in particular what role cell death plays in this process. Lymphocyte apoptosis has been observed in peripheral blood of patients with pneumococcal infection [12] and is a well-recognized feature of bacterial sepsis [13]. Nevertheless it remains unclear whether the lymphocyte apoptosis described during pneumococcal infection is part of a non-specific response, associated with microbial products and the altered cytokine responses that are a feature of infection, or whether it might be the result of a more specific host programme regulating the immune response.

We therefore examined whether the interaction of T-cells with pneumococci results in cell death and have characterized features of this process. In particular we observed that the pneumococcal protein pneumolysin induces T-cell necrosis but that in the presence of monocytes T-cells undergo Fas-dependent apoptosis. Moreover we have found a requirement for Fas-signaling in the regulation of CD3^+^ T-cell death, during the early T-cell dependent phases of the host response to pneumococci.

## Results

### Pneumococci induce T-cell apoptosis

**Figure 1:**
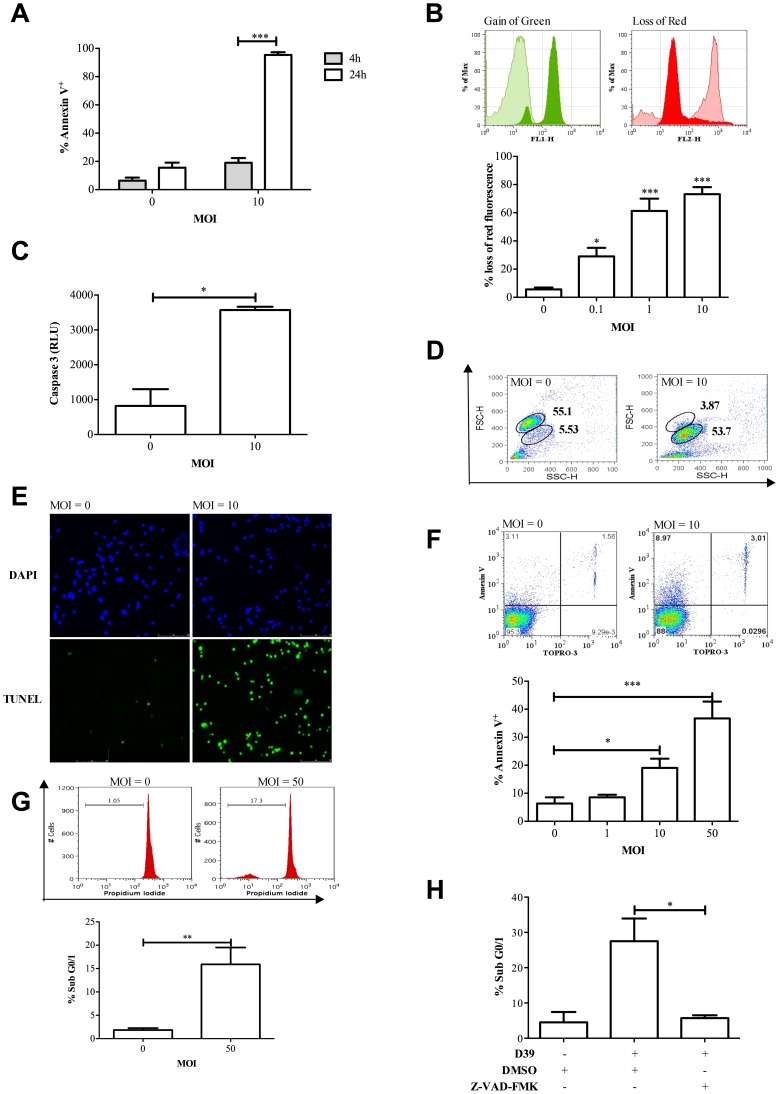
Peripheral blood mononuclear cells undergo apoptosis following challenge with Streptococcus pneumoniae. A) The percentage of Annexin V^+^ peripheral blood lymphocytes (PBL) identified by forward (FSC) and side scatter characteristics (SSC) 4 and 24 h after either mock-infection (multiplicity of infection (MOI) = 0) of peripheral blood mononuclear cells (PBMC) or challenge with D39 *Streptococcus pneumoniae* (MOI = 10), n = 6. B) Loss of inner mitochondrial transmembrane potential (Δψ_m_) was detected with the cationic dye JC-1 in PBL 16 h post challenge of PBMC (MOI = 0–10). Representative histograms demonstrate the gain of green and loss of red fluorescence following mock infection (tinted histogram, MOI = 0) or pneumococcal challenge (filled histogram, MOI = 10), the graph depicts the percent loss of red fluorescence indicative of loss of Δψ_m_, n = 6. C) Caspase 3 activation in PBMC 16 h post challenge (MOI = 0 or 10), recorded as caspase 3 relative luminescence units (Caspase 3 RLU), n = 3. D) Dot plots of the FSC and SSC profile of PBMC demonstrating a loss in FSC 16 h post challenge (MOI = 0 or 10), representative of three individual experiments. E) Representative images of PBMC 24 h following challenge (MOI = 0 or 10) and stained with DAPI and terminal UTP nick-end labeling (TUNEL). Results are typical of three independent experiments. F) A representative image showing Annexin V and TOPRO-3 staining is shown at MOI = 0 and 10 and the mean percentage Annexin V^+^ PBL gated by FSC and SSC after challenge of plastic purified PBMC (MOI = 0–50) for 6 h, n = 3. G) Representative histograms and graph of mean percentage CD3^+^ T-cells with accumulation of hypodiploid DNA (Sub G0/1) following challenge (MOI = 0 or 50) for 6 h. T-cells were identified by labeling with FITC conjugated anti-CD3, n = 4. H) The percentage Sub G0/1 CD3^+^ T-cells from PBMC pre-treated with either vehicle control (DMSO) or the pan-caspase inhibitor Z-VAD-FMK (10 µM) and mock-infected (D39−) or challenged with *S. pneumoniae* (D39+, MOI = 10) for 16 h, n = 3. Statistical analyses by ANOVA or t-test; * p<0.05, ** p<0.01, *** p<0.001.

We first examined whether peripheral blood mononuclear cells (PBMC) incubated with pneumococci demonstrated features of apoptosis, using a range of morphologic and biochemical assays of apoptosis. Almost all PBMC became Annexin V^+^ after 24 h of incubation with pneumococci (Figure 1A). PBMC also showed evidence of loss of inner mitochondrial transmembrane potential (**Δψ**_m_) (Figure 1B) and of increased caspase activation (Figure 1C), cell shrinkage (Figure 1D) and DNA strand breaks (Figure 1E), following exposure to pneumococci. We confirmed cell death of purified lymphocytes 6 h after exposure to bacteria with evidence of both early apoptotic Annexin V^+^/TO-PRO-3^−^ cells and late apoptotic/necrotic Annexin^+^/TO-PRO-3^+^ cells (data not shown), that was directly proportional to the MOI (Figure 1F). We also confirmed accumulation of hypodiploid DNA, a feature of apoptosis [14], in CD3^+^ T-cells (Figure 1G) and that the CD3^+^ T-cell death was caspase dependent (Figure 1H). The combination of features confirmed an apoptotic form of cell death. Apoptosis was apparent with an MOI as low as 0.1 (Figure 1B).

Cell death required cell contact between bacteria and PBMC and was not the consequence of utilization of growth factors in the media by bacteria since the insertion of transwells between bacteria and PBMC inhibited cell death at representative early and late time points after bacterial challenge (Figure S1). In PBMC cultures as expected T-cells predominated but cultures also contained 17.5±1.7% CD19^+^ B-cells and 8.7±1.1% CD14^+^ monocytes. CD19^+^ B-cells showed a trend towards an increased rate of cell death following 16 h pneumococcal challenge (Figure S2A). As we have previously reported during pneumococcal infection [15], CD14^+^ monocytes were highly susceptible to cell death (Figure S2B). We also confirmed that different subsets of T-cells were highly susceptible with early evidence of cell death (Figure S2C–E). These subsets included CD161^+^ cells, which are Th17 cells or cells with the potential to differentiate into this subset [16]. We did not find evidence for the selective involvement of any of these T-cell sub-sets as they were equally susceptible to cell death following bacterial challenge.

### Monocytes in cell cultures are required for the induction of CD3^+^ T-cell apoptosis

**Figure 2:**
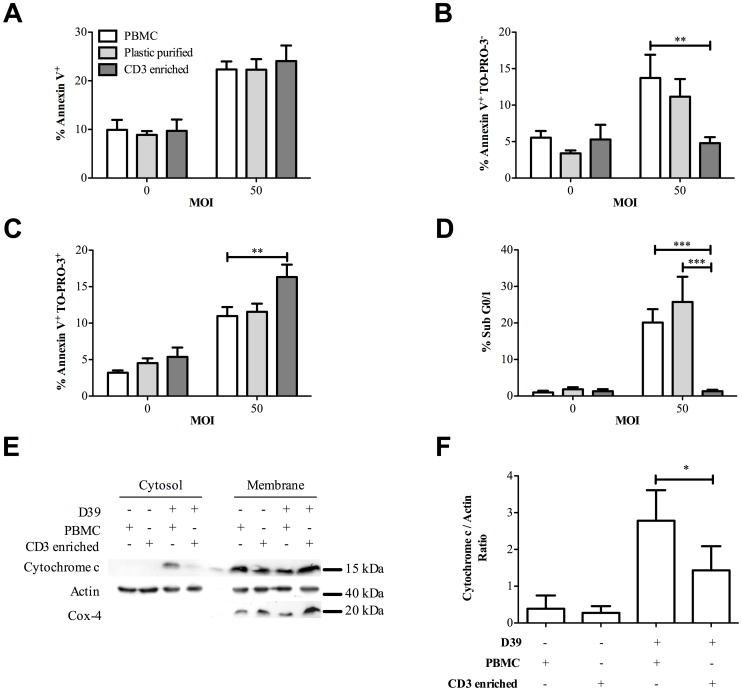
Monocytes induce CD3^+^ T-cell apoptosis following Streptococcus pneumoniae challenge. Peripheral blood mononuclear cells (PBMC), plastic purified lymphocytes (Plastic purified) and highly purified CD3^+^ T-cells (CD3 enriched) were challenged for 6 h with either mock-infection (multiplicity of infection (MOI) = 0) or D39 *Streptococcus pneumoniae* (MOI = 50). A) Total Annexin V^+^ B) early apoptotic, Annexin V^+^/TO-PRO-3^−^ and C) late apoptotic/necrotic Annexin V^+^/TO-PRO-3^+^ events were recorded, n = 11. D) Hypodiploid DNA (Sub G0/1) was measured in CD3^+^ T-cells under the same conditions, n = 5. E) PBMC and CD3 enriched T-cells were challenged with D39 (D39+) MOI = 10 or mock-infected (D39−) for 16 h. CD3^+^ T-cells were purified from the PBMC and cytosolic and membrane fractions were obtained from the CD3^+^ T-cells from each sample prior to western blot analysis probing for cytochrome c, actin and Cox-4 and F) densitometry summarises the cytochrome c/actin ratio for cytosols under each experimental condition, from four experiments with separate donors.

Since monocytes/macrophages can induce apoptosis in T-cells in specific circumstances [17], [18] we addressed whether monocytes influenced CD3^+^ T-cell death in cultures of PBMC following exposure to pneumococci. The percentage of CD14^+^ monocytes in PBMC was 11.8±1.1%, while the number in the non-adherent fraction of plastic purified PBMC cultures was reduced to 1.6±0.4% (Figure S3) and the percentage of CD14^+^ monocytes in highly pure CD3^+^ T-cells cultures was negligible at only 0.1±0.05%. In these experiments the highly pure CD3^+^ T-cell cultures contained 95±0.7% CD3^+^ T-cells. As shown in Figure 2A, the increase in total Annexin V^+^ cells, in PBMC cultures following challenge with pneumococci, was similar in magnitude despite varying numbers of monocytes. However, the PBMC cultures demonstrated significantly more Annexin V^+^/TO-PRO-3^−^ cells and significantly fewer Annexin V^+^/TO-PRO-3^+^ cells than the highly purified CD3^+^ T-cell cultures (Figure 2B–C), while purified CD3^+^ T-cell cultures showed no increase in Annexin V^+^/TO-PRO-3^−^ cells above baseline. Moreover few cells in the purified CD3^+^ T-cell culture showed accumulation of hypodiploid DNA or cytochrome c translocation into the cytoplasm, a specific feature of apoptosis indicating mitochondrial outer membrane permeabilization [19]. In contrast PBMC or plastic purified PBMC had significant numbers of cells with hypodiploid DNA and evidence of cytochrome c translocation (Figure 2D–F). To ensure cytochrome c translocation was measured only in CD3^+^ T-cells these cells were purified from PBMC cultures exposed to bacteria. We confirmed the lack of apoptotic features, such as hypodiploid DNA accumulation, in the purified CD3^+^ T-cell culture was not just the result of altered kinetics of cell death since the differences in accumulation of hypodiploid DNA between purified CD3^+^ T-cell cultures and PBMC were seen at all time points from 6–16 h after pneumococcal challenge, the latest time point being a time at which cell death was extensive in both cultures using less selective assays (Figure S4). The ability of monocytes to induce apoptosis in T-cells was not altered in the presence of neutrophils or apoptotic neutrophils, demonstrating this role of monocytes is not subverted during an acute inflammatory response or during the resolution of this response (Figure S5).

### CD14^+^monocytes reconstitute CD3^+^ T-cell apoptosis in cell cultures

**Figure 3:**
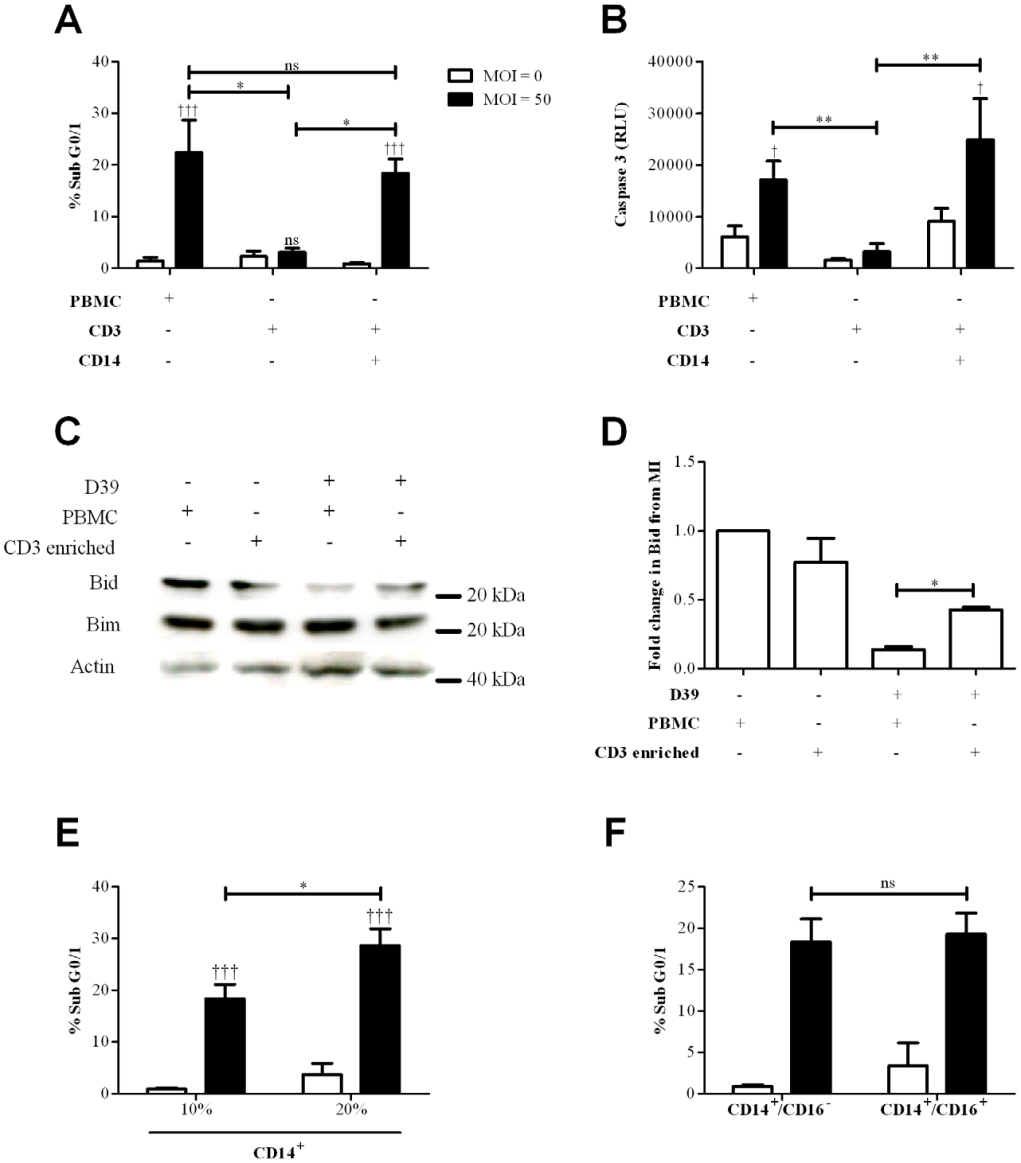
CD14^+^ monocytes reconstitute apoptosis in CD3^+^ T-cells following Streptococcus pneumoniae challenge. A) Peripheral blood mononuclear cells (PBMC), purified CD3^+^ T-cells (CD3^+^ CD14^−^) or co-cultures of purified CD3^+^ T-cells with 10% purified CD14^+^ monocytes (CD3+ CD14+) were mock-infected (MOI = 0, white bars) or challenged with D39 *Streptococcus pneumoniae* (MOI = 50, black bars) for 16 h and accumulation of hypodiploid DNA (% Sub G0/1) measured in CD3^+^ T-cells, n = 4. B) caspase 3 activation in CD3^+^ T-cells under the same condition as A), n = 4. C) Representative western blot probed for Bid, Bim and actin from CD3^+^ T-cells isolated from PBMC or CD3 enriched cultures following bacterial challenge as in A), D) densitometry summarizes the fold change in Bid compared to the mock infected (MI), derived from three separate experiments. E) % Sub G0/1 purified CD3^+^ T-cells in co-cultures containing 10% or 20% of purified CD14^+^ monocytes under the same conditions as A), n = 4 or in F) co-cultures of CD3^+^ T-cells and purified CD14^+^ monocytes with (CD14^+^/CD16^−^) or without (CD14^+^/CD16^+^) CD16^+^ monocyte depletion, under the same conditions as A), n = 4, ns (not significant) * p<0.05, ** p<0.01, *** p<0.001; statistical analysis by ANOVA.

To prove that monocytes were responsible for the enhanced levels of apoptotic T-cells we purified both CD3^+^ T-cells and CD14^+^ monocytes and confirmed that addition of monocytes to CD3^+^ T-cells was sufficient to induce accumulation of hypodiploid DNA and caspase 3 activation in CD3^+^ T-cells (Figure 3A–B). Apoptosis was not apparent in CD3^+^ T-cells cultured without CD14^+^ monocytes, even when cultured with high doses of bacteria for prolonged periods. Cultures containing CD14^+^ monocytes also showed evidence of a further apoptosis marker, loss of full length Bid indicative of Bid activation (Figure 3C–D). Bim expression remained constant in these experiments. In cultures containing only purified CD3^+^ T-cells there was no evidence of Bid activation. We were able to show that as the percentage of CD14^+^ monocytes increased the numbers of CD3^+^ T-cells with hypodiploid DNA increased (Figure 3E). Our isolation method initially only collected ‘classical’ CD14^+^ monocytes (Figure S6). ‘Non-classical’ CD14^lo^CD16^+^ monocytes have emerged as important effectors of innate immunity [2]. We therefore modified our isolation protocol to include ‘non-classical’ and also ‘intermediate’ populations [20], as shown in Figure S6. Addition of these sub-populations to the ‘classical’ monocytes did not alter the level of CD3 T-cells with hypodiploid DNA in comparison to co-cultures with only CD14^+^ monocytes (Figure 3F).

### In the absence of monocytes CD3^+^ T-cells undergo a form of toxin-mediated necrotic cell death

**Figure 4:**
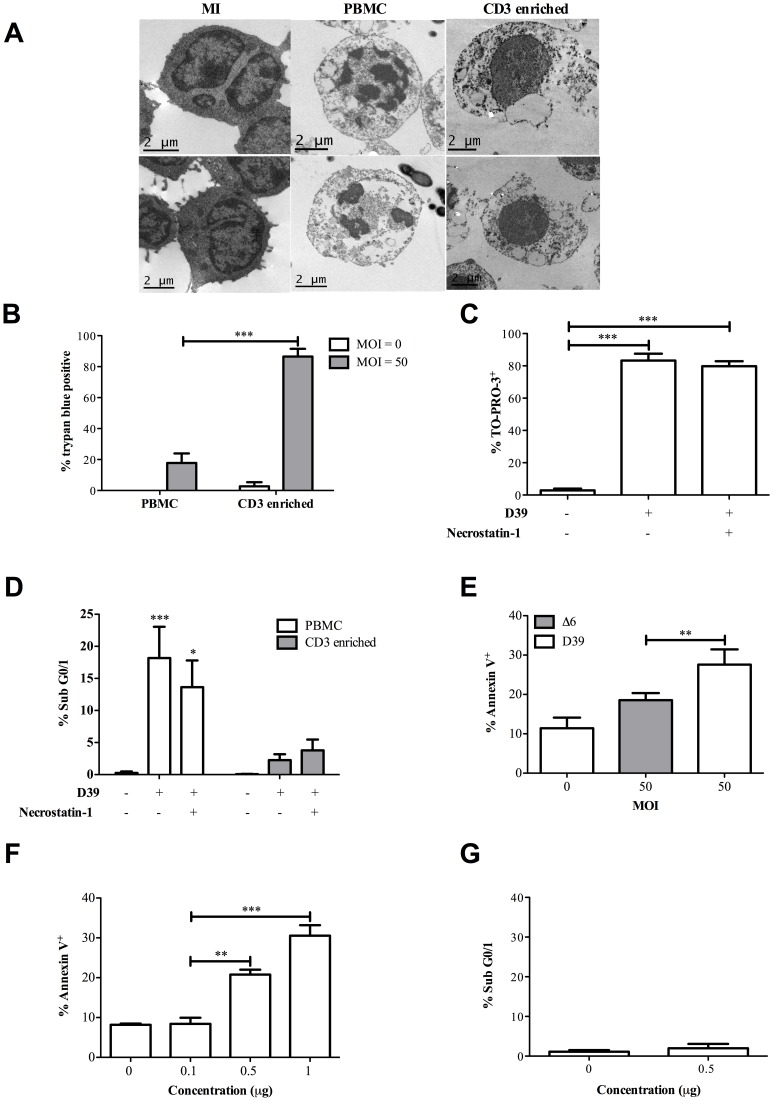
Purified T-cells undergo a necrotic pneumolysin-dependent death. A) Representative transmission electron micrograph of CD3^+^ T-cells from peripheral blood mononuclear cells (PBMC) and CD3 enriched cultures (CD3 enriched) following challenge with D39 *Streptococcus pneumoniae* (MOI = 50) or mock-infection (MI) for 6 h. CD3^+^ T-cells from PBMC cultures showed characteristics of apoptosis including nuclear fragmentation while CD3 enriched T-cells had features of necrosis such as membrane rupture. Images were obtained with an FEI Tecnai transmission electron microscope. B) T-cell trypan blue exclusion was assessed by brightfield microscopy following challenge of PBMC and CD3 enriched cultures with D39 (MOI = 50) for 6 h and C) pre-treatment with necrostatin-1 (30 nM) under the same conditions as B) did not reduce CD3^+^ death measured by TO-PRO-3 staining or D) convert the death process in CD3 enriched cultures to apoptosis, measured by accumulation of hypodiploid DNA (% Sub G0/1), n = 4. E) The mean percentage of Annexin V^+^ CD3^+^ T-cells 6 h after mock-infection (MOI = 0) or challenge of PBMC with D39 or the Δ6 mutant (MOI = 50), n = 5. F) The mean percentage of Annexin V^+^ and G) % sub G0/1peripheral blood lymphocytes isolated by plastic adherence of PBMC and gated by FSC and SSC 6 h following challenge with pneumolysin (0–1 µg), n = 3 *p<0.05, **p<0.01, ***p<0.001; statistical analysis by one and two-way ANOVA or t-test.

In the absence of monocytes CD3^+^ T-cells underwent a form of necrotic cell death. There was evidence of extensive loss of membrane integrity in the purified CD3^+^ T-cells and evidence of morphological features of necrosis such as prominent membrane disruption without features of cell shrinkage or of the nuclear condensation observed in the CD3^+^ T-cells isolated from PBMC (Figure 4A). Purified CD3^+^ T-cells showed prominent membrane disruption as evidenced by greater levels of Trypan blue staining (Figure 4B). The cell death in purified CD3^+^ T-cell cultures was not altered by necrostatin (Figure 4C) and death was not converted to apoptosis (Figure 4D) suggesting the death process did not involve necroptosis. Purified CD3^+^ T-cells did not demonstrate caspase 1 activation suggesting pyroptosis was unlikely to be the mechanism of cell death (Figure S7A). Cell death in PBMC required live bacteria (Figure S7B).

The pneumococcal virulence factor pneumolysin induces not only cell lysis with membrane permeabilization but also apoptosis in mammalian cells [21]. Distinct regions of pneumolysin govern cytolytic and non-cytolytic activity such as complement activation. Overall cell death in CD3^+^ T-cells in PBMC cultures, which we show above is apoptotic, was partially reduced but not restored to baseline by a mutant (**Δ**6), which produces toxin that lacks cytolytic activity (Figure 4E and Figure S7C). Purified pneumolysin could induce cell death of lymphocytes (Figure 4F) but these cells did not have features of apoptosis such as accumulation of hypodiploid DNA (Figure 4G). These results demonstrate that the purified T-cells die by a primarily necrotic process with prominent membrane permeabilization and implicate pneumolysin in the cell death.

### Monocytes enhance CD3^+^ T-cell activation during pneumococcal infection and increased activation enhances apoptosis

**Figure 5:**
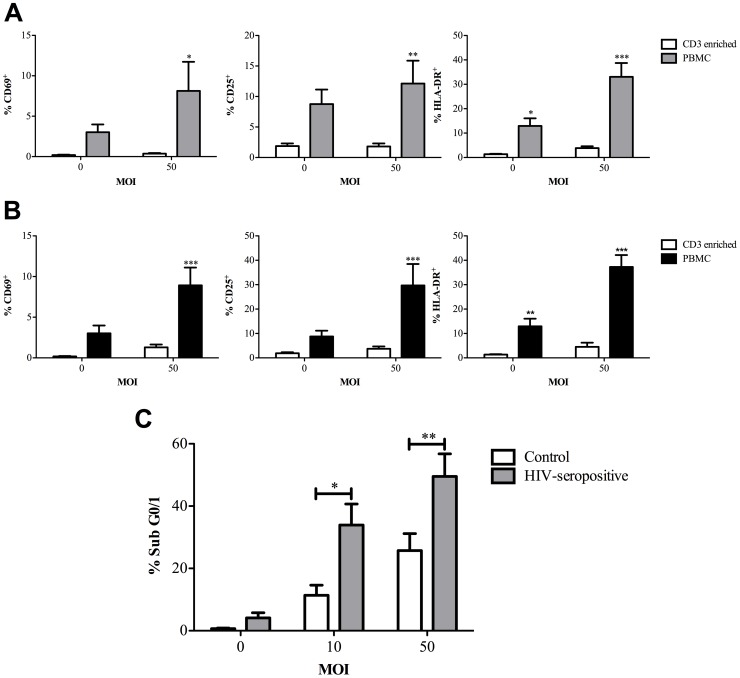
Monocytes enhance CD3^+^ T-cell activation following bacterial challenge and enhanced activation increases apoptosis. The percentage of CD3^+^ T-cells positive for CD69, CD25 and HLA-DR in highly purified CD3^+^ T-cells (CD3 enriched) or peripheral blood mononuclear cells (PBMC) following either mock-infection (multiplicity of infection (MOI) = 0) or challenge with A) live *Streptococcus pneumoniae* (MOI = 50) for 16 h, n = 8 or B) heat killed *S. pneumoniae* (MOI = 50) for 72 h, n = 4, * p<0.05, ** p<0.01, *** p<0.001; statistical analysis by ANOVA. C) The percentage of CD3^+^ T-cells with accumulation of hypodiploid DNA (Sub G0/1) 6 h following mock-infection (multiplicity of infection (MOI) = 0) or challenge with *Streptococcus pneumoniae* (MOI = 10 or 50) of peripheral blood mononuclear cells (PBMC) from control (n = 6) or HIV-seropositive individuals, n = 8, *p<0.05, **p<0.01; statistical analysis by ANOVA.

We next addressed whether monocytes altered CD3^+^ T-cell activation in our model, since monocytes can enhance MHC-independent T-cell activation [22] and enhanced activation can alter susceptibility to apoptosis [23]. Monocytes/macrophages can also induce death receptor mediated cell death in activated T-cells [18]. We showed enhanced expression of the CD3^+^ T-cell activation markers CD69, CD25 and HLA-DR in PBMC cultures exposed to viable or heat-killed pneumococci (Figure 5A–B). CD3^+^ T-cells from HIV-1 seropositive individuals are known to have enhanced levels of activation and to be particularly susceptible to macrophage-mediated cell death [18]. In keeping with this we documented that cells isolated from HIV-seropositive individuals who were not receiving antiretroviral therapy (since HIV viral load influences activation state and susceptibility to apoptosis [24]), had increased levels of CD3^+^ T-cell death, as compared to controls, when exposed to pneumococci (Figure 5C). These results suggested T-cell activation influenced levels of cell death and activation was enhanced in PBMC co-cultures containing monocytes.

### CD3^+^ T-cell apoptosis is Fas-mediated in PBMC during pneumococcal infection

**Figure 6:**
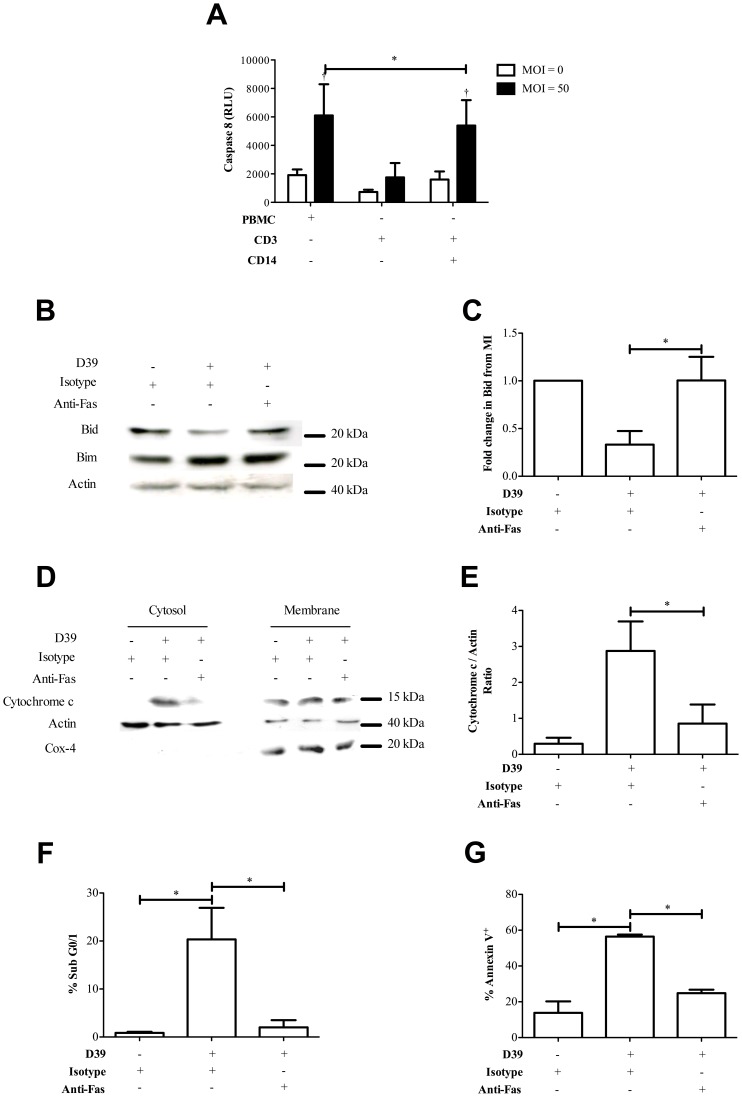
CD3^+^ T-cells undergo Fas-mediated apoptosis following pneumococcal challenge. A) Mean caspase 8 relative luminescence units (Caspase 8 RLU) in CD3^+^ T-cells from peripheral blood mononuclear cells (PBMC), purified CD3^+^ T-cells (CD3+ CD14−) or co-cultures of purified CD3^+^ T-cells with 10% purified CD14^+^ monocytes (CD3+ CD14+) 6 h following mock-infection (multiplicity of infection (MOI) = 0) or challenge with D39 *Streptococcus pneumoniae* (MOI = 50), n = 5. B) Representative western blot probed for Bid, Bim and actin from CD3^+^ T-cells purified from PBMC 16 h following mock-infection (D39−) or challenge with D39 at MOI = 0.1 (D39+) in the presence of isotype control (Isotype+) or ZB4 neutralizing anti-Fas antibody (Anti-Fas+) added at 1 µg/ml. C) Densitometry summarises the fold change in Bid compared to the mock infected (MI) cells from three separate experiments with different donors. D) Cytosolic and membrane fractions were also probed for cytochrome c, actin and Cox-4, and E) densitometry was performed, n = 4. F) Hypodiploid DNA accumulation (Sub G0/1) was measured in CD3^+^ T-cells in PBMC cultured under the same conditions as B), n = 4. G) The percentage of Annexin V^+^ CD3^+^ T-cells in PBMC cultured under the same condition as in B), n = 3, * p<0.05; statistical analysis by ANOVA or t-test.

To determine the apoptotic pathway inducing monocyte-dependent CD3^+^ T-cell apoptosis we next examined the potential role of Fas ligand (FasL). Fas signaling contributes to T-cell receptor mediated activation-induced cell death but has also been implicated in macrophage-induced cell death of activated T-cells [18], [23]. PBMC were challenged with pneumococci and then CD3^+^ T-cells were purified to prevent other mononuclear cells from confounding the results. This demonstrated increased levels of caspase 8 activation in CD3^+^ T-cells co-cultured with monocytes (Figure 6A), suggesting death receptor-mediated apoptosis [23]. We next examined involvement of Fas in the death process using an infectious dose at the low end of the range we had shown causes death (see Figure 1B) to limit competing effects of necrosis. A Fas blocking antibody prevented Bid activation and cytochrome c accumulation in the cytosol of CD3^+^ T-cells present in PBMC (Figure 6B–E). The numbers of CD3^+^ T-cells with accumulation of hypodiploid DNA was also reduced following treatment with anti-Fas antibody (Figure 6F). In addition, when we supplied a Fas-blocking antibody and measured the level of Annexin V^+^ cells in CD3^+^ T-cells isolated from PBMC following pneumococcal infection, there was also a reduction of cell death (Figure 6G). Similar experiments in purified CD3^+^ T-cells revealed Fas inhibition failed to reduce the levels of Annexin V^+^ CD3^+^ T-cells (data not shown). In addition, a granzyme B inhibitor (added since it has been suggested that pneumolysin can substitute for perforin and combine with granzyme B to induce lymphocyte apoptosis [25]) did not alter levels of T-cell apoptosis (Figure S8). TRAIL blocking antibody or PDL-1 blocking antibody also had no effect on levels of T-cell apoptosis (data not shown). These results confirmed that Fas mediated CD3^+^ T-cell apoptosis in PBMC cultures.

### Evidence of Fas-mediated T-cell death and a requirement for Fas ligand during invasive pneumococcal disease

**Figure 7:**
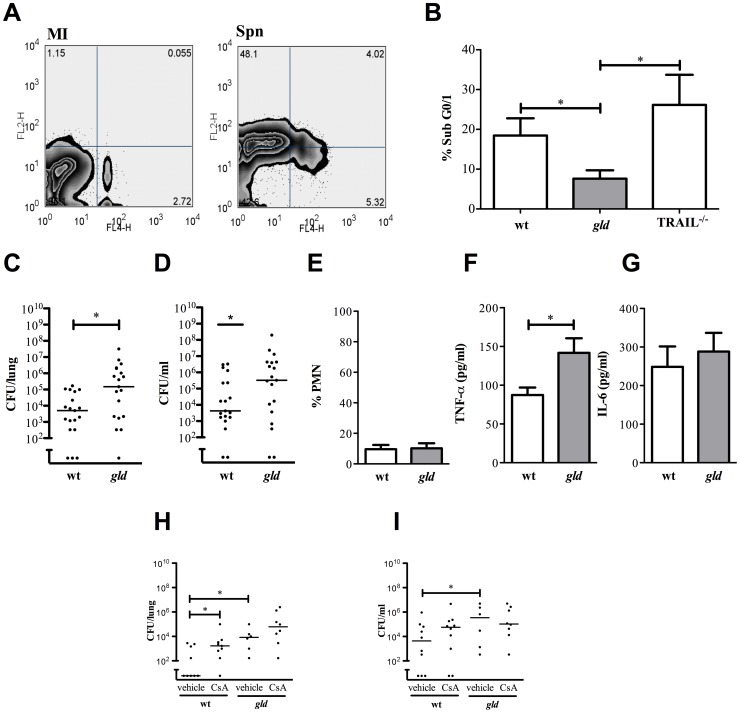
FasL regulates T-cell apoptosis and bacterial clearance in a murine model of invasive pneumococcal disease. A) Representative zebra plots measuring cell death in C57BL/6 mouse CD3+ T-cells isolated from spleens 24 h after mock-infection (MI) or intra-tracheal instillation with serotype 1 Streptococcus pneumoniae (Spn) (1×107 cfu). The zebra plots show Annexin V+ (FL2-H) and TO-PRO-3+ (FL4-H) staining and the percentage of cells in each quadrant. B) The percentage CD3+ T-cells with accumulation of hypodiploid DNA (% Sub G0/1) isolated from spleens of mice 24 h after instillation of serotype 4 S. pneumoniae (1×107 cfu) to C57BL/6 wild-type (wt), generalized lymphoproliferative disease (gld) and TNF-related apoptosis inducing ligand deficient (TRAIL−/−) mice. n = 5, * p<0.05; statistical analysis by t-test. C) Colony forming units (CFU) of bacteria in lung homogenates from wt and gld mice 24 h after intra-tracheal instillation of 5×105 CFU serotype 4 pneumococci and D) CFU of bacteria in blood. E) Percentage of neutrophils (%PMN) in bronchial alveolar lavage in the same experiments as C) and F) levels of TNF-α and G) IL-6 in bronchoalveolar lavage fluid, n = 6. H) CFU of bacteria in lung homogenates from wt and gld mice 24 h after intra-tracheal instillation of 5×105 CFU serotype 4 pneumococci after treatment with cyclosporine A (CsA). I) CFU of bacteria in blood in the same experiments as H). Statistical analysis by t-test or Kruskal-Wallis test with Dunn's Multiple Comparison Test, * p<0.05.

To confirm the relevance of these findings *in vivo* we documented T-cell apoptosis in the spleen, an environment where macrophage-mediated T-cell death would be anticipated to occur *in vivo* [26], in mice with invasive pneumococcal disease (Figure 7A). Differences in levels of bacteremia can influence levels of T-cell apoptosis [27]. Therefore our investigation of apoptosis *in vivo* was carried out using a high dose of serotype 4 *S. pneumoniae*, a virulent strain of bacteria that establishes high levels of bacteremia [28], which we confirmed were similar between FasL deficient *gld* and control mice (data not shown). Under these conditions, which normalized blood bacterial colony counts, we documented a significant reduction in the levels of hypodiploid DNA in CD3^+^ T-cells from *gld* mice, as compared to TRAIL deficient or wild-type mice (Figure 7B).

However, T-cells play an important role in the early stages of pneumonia [5], [6]. To test the relevance of FasL to pneumonia outcomes we confirmed that *gld* mice had increased levels of bacteria in the lung and blood in a model when there were very low levels of recruited neutrophils (Figure 7C–E). In association with this *gld* mice had increased levels of TNF-α (Figure 7F), though not IL-6 (Figure 7G). We also established that T-cells in this model play an important role in host defense by establishing that control mice treated with cyclosporine, a known T-cell immunosuppressant and cause of T-cell lymphopenia [11], had reduced control over bacterial replication and that this T-cell mediated control was lost in the *gld* deficient mice (Figure 7H–I). This suggested FasL was critical for T-cell dependent host defense in the early stages of pneumococcal infection when there are still relatively few recruited neutrophils.

## Discussion

Monocytes are usually regarded as contributing to the ramp up of inflammatory responses rather than triggering their cessation [2]. We now demonstrate an important role for monocytes in controlling the number of activated T-cells during bacterial infection. Although a variety of T-cell populations contribute to the immune response to bacteria such as pneumococci [5], [7], [10], the regulation of these cell populations and the role of cell death in this process are incompletely defined. We show that monocytes play an important and unrecognized role ensuring Fas-dependent apoptosis to ensure the safe disposal of activated T-cells. In the absence of monocytes pathogen driven T-cell necrosis predominates. Moreover we also demonstrate that in mice T-cell dependent early host responses to pneumococcal infection are attenuated in the absence of FasL, suggesting that monocyte-mediated Fas-dependent apoptosis represents a critical component of the early stages of an effective host response against pneumococci. We were also able to show this response could occur in the presence of viable or apoptotic neutrophils, suggesting it may also be important at later stages of infection.

Cholesterol-dependent cytolysins, including listeriolysin, are recognized stimuli for T-cell death [27]. We found the related pneumococcal toxin pneumolysin triggered non-apoptotic death with early membrane permeabilization in purified T-cells. Pneumolysin enhanced monocyte-dependent T-cell death, though in this case via apoptosis without evidence of early membrane disruption, suggesting apoptosis did not require outer cell membrane pore formation. Cytolytic activity of pneumolysin has been traditionally viewed as a virulence determinant but recent evidence suggests that pneumolysin is also an important trigger for effective innate immune responses against the pneumococcus, including those mediated by the nucleotide-binding oligomerization domain-like receptor family pyrin domain containing 3 (NLRP3) inflammasome [29]. In our model of monocyte-mediated apoptosis it appears that the requirement for cytolytic toxin was as a trigger for the host response that induced maximal levels of apoptosis. Mutants that lack cytolytic activity such as ST306 serotype 1 and ST53 serotype 8 pneumococci have emerged as major causes of invasive pneumococcal disease [30]. On the basis of our findings we would predict these strains would induce less T-cell apoptosis and that persistence of activated T-cells could contribute to the invasive potential of these non-cytolytic strains. In contrast, once these strains have invaded the propensity to cause lower rates of apoptosis might explain the lower mortality associated with them since lymphocyte apoptosis may influence outcomes during sepsis [31]. Surprisingly PBMC needed to be in contact with the bacteria for the toxin to mediate its effect. Although the toxin is soluble when released from lysed bacteria it may also remain associated with the bacterial cell wall [32], which might explain this finding. Alternatively cell contact with PBMC might upregulate toxin production or enhance release by increasing bacterial lysis [33].

LPS can stimulate MHC-independent T-cell activation via a monocyte-dependent process involving CD80 [22]. Pneumolysin is also a TLR4 ligand that activates T-cells; therefore a similar mechanism may explain the enhanced T-cell activation we observed in PBMC cultures [5], [21], although the fact that heat killed bacteria also activated T-cells suggests active toxin is not essential. Other microbial factors could also stimulate monocytes to activate CD3^+^ T-cells. One consequence of increased T-cell activation is to enhance susceptibility to apoptosis after a period of sustained activation and this paradigm is exemplified by the apoptosis of uninfected CD3^+^ T-cells during HIV infection [34]. In keeping with this PBMC from HIV-seropositive individuals had elevated levels of CD3^+^ T-cell apoptosis following challenge with pneumococci. In addition to enhancing susceptibility to apoptosis T-cell activation could increase resistance to the competing toxin-related cytolytic cell death through cytokine production. For example, IFN-γ, produced by activated Th1 cells, reduces the susceptibility of epithelial and monocytic cell lines to pneumolysin associated cytolytic death [35]. Consistent with this possibility mice lacking IFNγ are more susceptible to listeriolysin dependent cell death [27].

In the presence of ‘classical’ monocytes we found that CD3^+^ T-cell death had classic features of apoptosis and this effect was maintained even in the presence of small numbers of monocytes, as evidenced by high levels of CD3^+^ T-cell apoptosis in plastic purified PBMC. ‘Non-classical’ monocytes played less of a role in this process though the numbers we isolated were relatively small so we cannot exclude a possible role when their numbers expand. The absence of early membrane permeabilization or of inhibition by necrostatin argued against the cell death being a form of programmed necrosis and the absence of cell swelling, early loss of membrane integrity or caspase 1 activation was inconsistent with pyroptosis, which has recently been described in T-cells [36]. In contrast cell death in purified CD3^+^ T-cells which were not co-cultured with monocytes was characterized by early membrane disruption without significant mitochondrial outer membrane permeabilization, Bid activation or accumulation of hypodiploid DNA, consistent with necrosis.

These results define a novel role for monocytes in downregulating the inflammatory response during acute bacterial infection. A key role for monocytes in downregulating the inflammatory response has been previously ignored. Unlike macrophages, monocytes are not thought to clear apoptotic cells by efferocytosis [3]. Nevertheless, monocytes express FasL [37] and evidence that monocytes induce Fas-mediated apoptosis of activated T-cells has been provided during HIV infection [17] and chronic periodontitis [38]. Nevertheless this role of monocytes has not been demonstrated previously during acute bacterial infections, where downregulation of the inflammatory response is a critical component of inflammation resolution. In our model monocytes did not influence overall T-cell viability (levels of death were similar in the presence or absence of monocytes) but their role was to ensure the induction of an injury limiting apoptotic form of cell death. There are a number of potential benefits to the host of monocyte-dependent CD3^+^ T-cell apoptosis: ensuring the timely removal of activated T-cells downregulates pro-inflammatory cytokine expression by T-cells, induction of apoptosis rather than necrosis limits secondary tissue injury and the removal of apoptotic cells by efferocytosis further detunes pro-inflammatory cytokine expression by macrophages during pneumococcal pneumonia [39]. Consistent with these possibilities we noted that FasL deficient mice had reduced T-cell apoptosis (when we normalized the level of bacteraemia to exclude confounding effects of differences in bacterial number) and that during the early T-cell dependent stages of pneumococcal pneumonia FasL deficiency was associated with impaired bacterial clearance, in a model that had few recruited neutrophils and was therefore unlikely to be confounded by any direct effects of Fas-signalling on neutrophil function [40]. FasL deficiency was associated with upregulation of TNF-α which would be anticipated to in turn upregulate receptors involved in the translocation of bacteria into the blood such as the PAF receptor [41]. Activated T-cells enhance TNF-α production by monocytes, through release of IFN-γ, but also through membrane interactions with monocytes [42], [43]. Induction of T-cell apoptosis not only would be anticipated to reduce these stimuli but would also provide apoptotic cells which when ingested by macrophages would further reduce TNF-α expression [39]. IL-6 production, however, was not reduced even though production is also decreased in the presence of apoptotic cells [39]. The reason for this finding was not apparent but may reflect the pleiotropic role of IL-6 both as an inflammatory cytokine and as a cytokine which can downregulate T-cell responses [44]. It remains possible that its production was sustained since it is produced as part of a counter-regulatory response to the sustained presence of activated T-cells [44], although we were not able to formally test this possibility.

Recently it has been shown that selective CD4^+^ T-cell depletion, non-selective depletion of T-cells or reduction of T-cell activation can improve survival in murine models of invasive pneumococcal disease, suggesting excessive T-cell activation can be harmful and must be tightly controlled [11]. Our observations provide a mechanism for this process. Prior observations of T-cell apoptosis and of increased FasL expression in a small cohort of patients with pneumococcal disease are also consistent with this [12]. We established a hierarchy of cell death programs such that monocytes induced apoptosis as the dominant mechanism of T-cell death during pneumococcal infection. In the absence of monocytes the combination of non-apoptotic cell death and the unopposed effects of pro-inflammatory microbial factors, such as pneumolysin, would be predicted to result in increased tissue injury and bacterial invasion [21]. In the context of pneumolysin-mediated T-cell activation [5], and in light of the potential for pneumococci to induce a predominantly cytolytic cell death in purified CD3^+^ T-cells, our observation that monocytes regulate T-cell death, ensuring Fas-mediated apoptosis predominates, is significant. These findings emphasise the important and unrecognised role of monocytes in downregulating the inflammatory response to bacterial infection by regulating numbers of activated T-cells during the immune response to infection.

## Materials and Methods

### Ethics

Human PBMCs were isolated from whole blood donated by healthy volunteers as previously described with written informed consent as approved by the South Sheffield Regional Ethics Committee of Royal Hallamshire Hospital (Sheffield, United Kingdom). All animal experiments were performed in accordance with the UK Animals (Scientific procedures) Act, authorized under a UK Home Office License, and approved by the animal project review committee of the University of Sheffield.

### Bacteria

Experiments were performed with serotype 2 S. pneumoniae, strain D39 (NCTC7466) or its Δ6 mutant unless otherwise stated. In murine experiments serotype 1 (SSISP strain; Statens Seruminstitut) or serotype 4 *S. pneumoniae* (TIGR4 strain) were utilized. To test the contribution of the pneumococcal endonuclease EndA [45] in increasing the extent of cleavage of hypodiploid DNA we screened a panel of serotype 1 strains for EndA production using the method of Beiter et al [45] and used strain NCTC7465 that is EndA negative and INV104B that expresses EndA. All bacterial stocks were prepared as described previously [46]. In certain experiments bacteria were heat-killed by boiling for 10 min prior to incubation with cells and inhibition of growth was confirmed by documenting no growth following plating on blood agar.

### Construction of D39Δ6

The **Δ**6 mutation refers to deletion of amino acid residues A146 and R147 from pneumolysin, introduced by site-directed mutagenesis [47]. The altered gene replaced the chromosomal gene [48]. The Janus cassette, which encodes markers for kanamycin resistance and streptomycin sensitivity, was linked to 300 bp of upstream and downstream flanking DNA from the pneumolysin gene by splice overlap PCR [48]. This construct was transformed into a streptomycin resistant version of D39. Homologous recombination in the flanking DNA introduced the Janus cassette in place of the pneumolysin gene giving an intermediate form of D39 that was resistant to kanamycin and sensitive to streptomycin. The intermediate form was isolated by growth on plates containing 150 mg/ml kanamycin and transformed with the **Δ**6 gene. Homologous recombination in the flanking DNA replaced the Janus cassette with the altered form of the pneumolysin gene giving D39 that was resistant to streptomycin and sensitive to kanamycin. Recombinants were selected by growth on plates containing 300 mg/ml streptomycin. The selected strain was checked for production of pneumolysin by western blot and the toxin produced was shown to be non-haemolytic using a standard hemolytic assay of cell extracts as described previously [49], see Figure S7C.

### Cell culture

Peripheral blood mononuclear cells (PBMC) plated at 1×10^6^ cells/ml were isolated and cultured as previously described [50]. Plastic purified lymphocytes (PBL) were obtained by plating PBMC at 2×10^6^ cells/ml in 25 cm^2^ flasks (Costar) for 1 h, transferring the non-adherent cells to a new flask for a further 1 h, removing, washing and counting the non-adherent cells and then resuspending at 1×10^6^ cells/ml in a 24-well plate. Purified CD3^+^ T-cells and monocytes with and without CD16 depletion were isolated from PBMC by magnetic immunoselection using EasySep human T cell enrichment kit, EasySep human monocyte enrichment kit and EasySep human monocyte enrichment kit without CD16 depletion (Stemcell Techonologies). In the absence of CD16 depletion monocyte sub-populations corresponding to ‘non-classical’ and ‘intermediate’ monocytes, as described by Ziegler-Heitbrock and colleagues [20] were identified (Figure S6). Neutrophils were isolated by dextran sedimentation and plasma-Percoll (Sigma) density gradient from the peripheral blood of healthy volunteers and were approximately 98% pure. Neutrophils were made apoptotic as previously described [51]. In brief neutrophils were cultured for 20 h to ensure a population that was approximately 80% apoptotic and <5% necrotic, as defined by trypan blue. Neutrophils immediately after isolation or apoptotic neutrophils were added to PBMC cultures at a ratio of 10 neutrophils per PBMC for the indicated periods.

### Infection of PBMC

Freshly isolated PBMC, plastic purified lymphocytes and CD3 enriched T-lymphocytes or cultures of purified CD3^+^ T-lymphocytes and purified monocytes were infected with *S. pneumoniae* or mock-infected for 4–24 h. In certain experiments transwells with 0.4 µm pore size (BD Biosciences) were used to separate bacteria from lymphocytes [52]. To inhibit caspase activation 10 µM of *N*-benzyloxycarbonyl-Val-Ala-Asp (*O*-methyl) fluoromethyl ketone (Z-VAD-FMK) (R&D Systems Inc.) or DMSO (Sigma) as vehicle control was added for 30 min before infection. ZB4 neutralizing Fas antibody at 1 µg/ml (Enzo Life Sciences), or isotype control, was added to cultures 30 min prior to infection [18]. In additional blocking experiments, cells were pre-treated for 30 min with 2.5 µg/ml of neutralizing TRAIL antibody 2E5 (Enzo Life Sciences), 10 µg/ml of mouse anti-human CD274 (PD-L1) (MIH1, eBioscience), 30 nM of necrostatin-1 (Sigma) to inhibit necroptosis, or 5 µM of granzyme B inhibitor (Calbiochem). In experiments involving antibodies monocyte Fcγ receptors were pre-blocked with 100 µg of human IgG_1_ (Sigma).

### Fluorescence microscopy

Cytospin preparations were generated from non-adherent PBMC after infection (Cytospin 3; Thermo Shandon) and slides stained with TUNEL (terminal deoxynucleotidyl transferase dUTP nick end labelling) reagents per the manufacturer's instructions (Oncor), counterstained with 4′6′-diamidino-2-phenylindole (DAPI (Vectashield)) and mounted with cover slips containing Vectashield. TUNEL and DAPI-positive cells were viewed via fluorescence light microscopy (Leica DMRB 1000).

### Transmission electron microscopy

PBMC or CD3 enriched T-cells were challenged with *S. pneumoniae* (serotype 2, D39) or mock-infected for 6 h then fixed in ice-cold 3% glutaraldehyde/0.1 M phosphate buffer overnight at 4°C. The cell pellets were processed as previously described [15]. Sections (85 nm) were cut on a Reichert Ultracut E ultramicrotome and stained with 1% toluidine blue in 1% borax. Sections were examined using an FEI Tecnai transmission electron microscope at an accelerating voltage of 80 kV and micrographs were taken using a Gatan digital camera.

### Flow cytometry

Flow cytometric measurements were performed using a four-colour FACSCalibur flow cytometer (Becton Dickinson). Forward and side scatter light was used to identify cell populations by size and granularity. Fcγ receptor blockade was with 100 µg/ml human IgG_1_ (Sigma). Cell surface marker expression was with 1 µg/ml mouse anti-human anti- CD14 (61D3) phycoerythrin (PE), (eBioscience), anti-CD3 (SK7) fluorescein isothiocyanate (FITC), (BD Pharmingen), anti-CD161 (HP-3G10) allophycocyanin (APC), (eBioscience), anti-CD4 (CSK3) peridinin chlorophyll protein (PerCP) and anti-CD19 (HIB19) FITC (BD Pharmingen) with appropriate isotype controls. To determine percentages of monocyte sub-populations after each isolation procedure we used 1 µg/ml mouse anti-human anti-CD14 (TuK4) pacific blue (Invitrogen) and anti-CD16 (B73.1) PE (eBioscience) or appropriate isotype controls. Activation marker expression was with 1 µg/ml of mouse anti-human anti-CD69 (FN50) APC (eBioscience); anti-human anti-CD25 (M-A251) APC-H7 (eBioscience) and anti-human anti-HLA-DR (L243) PE (BD Pharmingen). In murine experiments T-lymphocytes were identified using rat anti-mouse anti-CD3 (17A2) eFluor 450 (eBioscience). Annexin V-PE and TO-PRO-3 was used to assess cell death. Annexin V^+^/Topro3^−^ cells were regarded as early apoptotic and Annexin V^+^/TO-PRO-3^+^ cells as late apoptotic/necrotic [46]. To measure loss of the inner mitochondrial transmembrane potential (Δψ_m_), the cationic dye JC-1 was used. Loss of Δψ_m_ was confirmed by loss of red and gain of green fluorescence. DNA fragmentation was confirmed by the hypodiploid peak assay [14]. Apoptotic cells were visible as a distinct population with low fluorescence compared to healthy cells in growth phase 0/1. Since the events recorded were predominantly on the far left of the hypodiploid peak when depicted on a linear scale, we have presented the histograms on a log scale [53]. We tested the role of the pneumococcal endonuclease EndA in further degrading the DNA of the apoptotic cells [45]. Using pneumococcal strains that are sufficient in functional EndA, or lack significant EndA activity, we confirmed that EndA increased the degree of DNA degradation, pushing events to the left on a log scale and that this appearance could be reproduced by incubating apoptotic cells with 1 µg/ml of DNAse (Promega) (Figure S9). In all flow cytometry experiments 10,000 events were captured and analyzed with FlowJo software version 9.3.2 (Tree Star, Inc.).

### Caspase 3 and 8 activation

Caspase 3 and 8 activities were measured using the Caspase-Glo 3/7 Assay (Promega, USA) and Caspase-Glo 8 Assay (Promega, USA). After challenge with D39 (16 h, MOI 10), 4×10^4^ PBMC (or in some experiments CD3^+^ T-cells isolated from the PBMC by magnetic immunoselection as above) were suspended in 50 µl of media and were combined with 50 µl of the Caspase-Glo 3/7 or Caspase-Glo 8 reagent for 1 h at room temperature. Luminescence was measured with a Packard Bioscience Fusion universal microplate analyser (Perkin Elmer, Beaconsfield, UK).

### Western blot analysis

Cells were lysed, quantified and separated by SDS-PAGE as described with 15–40 µg protein/lane [49]. Proteins were transferred to Immobilon-P membrane (Millipore), blocked in PBST/5% non-fat dry milk powder and incubated with primary and secondary antibodies. Antibodies used were: mouse anti-human cytochrome c (7H8.2C12, BD Biosciences), mouse anti-human Cox-4 (Molecular Probes), rabbit anti-human Bid (Cell Signalling), rabbit anti-human Bim (Chemicon Ltd.), rabbit anti-human caspase 1 (Abcam) and actin (A2086, Sigma-Aldrich). Detection was with HRP-conjugated goat anti-rabbit and anti-mouse immunoglobulins (Dako), and enhanced chemiluminescence (Amersham Pharmacia).

### PBMC from HIV seropositive individuals

PBMC were isolated from 8 HIV-seropositive individuals who were not receiving antiretroviral therapy. Individuals had CD4 T-cell counts of 498±49/µl (Mean ± SEM) and HIV viral loads of 42379±15823 as assessed by Roche Cobas Ampliprep/Cobas TaqMan HIV-1 version 2.0. PBMC were challenged with pneumococci and apoptosis in CD3^+^ T-cells was measured by estimating hypodiploid DNA accumulation as above.

### Murine model of invasive pneumococcal disease and isolation of splenocytes

C57BL/6 mice (Harlan, Oxford, U.K.), mice homozygous for the FasL^gld^ mutation (B6Smn.C_3_-Fasl^gld^/J, *gld*) [54] and TRAIL deficient mice [55] both on a C57BL/6 background, received 5×10^5^ or 1×10^7^ cfu serotype 1 or 4 *S. pneumoniae* by intratracheal instillation for 24 h as described [46]. Excised spleens were homogenized and splenocytes separated from tissue using a 100 µm cell strainer (BD biosciences) before staining and analysis by flow cytometry. Bacterial colony counts were estimated in lung and blood as previously described [46]. Bronchoalveolar lavage (BAL) was performed by instilling 5×0.8 ml of ice-cold heparinized-saline intratracheally (Leo laboratories) and total cell counts estimated by hemocytometer [46]. Cytospins were prepared (Cytospin 3, Thermo Shandon) and the percentage neutrophils in the BAL estimated as described [46]. TNF-α and IL-6 were measured by EIA as described [39].

### Statistics

Results were recorded as mean and standard error of the mean with the number of individual donors cells contributing to each data set shown as the ‘n’ value. Differences between groups of treatments were calculated by ANOVA (Bonferroni's post-test), with Kruskal-Wallis test with Dunn's Multiple Comparison Test for non-parametric comparisons or with paired t-test for comparison of means using GraphPad Prism 5 (Graphpad Software, Inc.). Significance was defined as p<0.05.

## Supporting Information

Figure S1 **Cell contact is required for lymphocyte death.** Peripheral blood lymphocytes (PBL) were isolated by plastic adherence and mock infected (MOI = 0) or challenged with *Streptococcus pneumoniae* serotype 2 (D39). (MOI = 0.1–50) in the presence (TW+) or absence (TW−) of semi -permeable transwell membranes. A) PBL were challenged for 6 h, MOI = 0, 1, 10 or 50 or B) 24 hours, MOI = 0, 0.1, 1 or 10. Cell death was recorded by flow cytometry in lymphocytes gated on forward light scatter and side light scatter characteristics and stained with Annexin V and TOPRO-3. All Annexin V positive (Annexin V^+^) cells were treated as dead, n = 3, *** p<0.001, two-way ANOVA with Bonferroni post-test.
(TIFF)

Figure S2 **Sub-sets of peripheral blood mononuclear cells are susceptible to cell death following pneumococcal challenge.** The percentage Annexin V^+^ cells in subsets of peripheral blood mononuclear cells was estimated after mock-infection (MOI 0) or challenge with *Streptococcus pneumoniae* (MOI = 10 or 50 as indicated) and staining with anti -CD19, anti-CD14, anti-CD3 plus anti-CD4 or anti -CD3 plus anti -CD161. A) CD19^+^ B-cells after 16 h challenge, and B) CD14^+^ monocytes after 6 h challenge, C) CD3^+^/CD4^+^ T-cells and D) CD3^+^/CD4^+^ T-cells after 6 h challenge (all MOI = 50) and E) the percentage CD3^+^/CD161^+^ T-cells with hypodiploid DNA (Sub G0/1) after 16 h challenge (MOI = 10). n = 4, * p<0.05, *** p<0.001 statistical analysis by t -test.
(TIFF)

Figure S3 **CD14^+^ contaminating monocytes are effectively removed by CD3^+^ T-lymphocyte magnetic immunoselection.** Levels of CD14^+^ monocytes were measured in peripheral blood mononuclear cell (PBMC) populations subjected to various purification methods. PBMC, plastic purified lymphocytes (Plastic purified) and PBMC highly purified by magnetic immunoselection to yield CD3^+^ T-lymphocytes (CD3 enriched) at high purity were stained with mouse anti-human CD14 or isotype, and analysed by flow cytometry. A) Representative dot plots and B) a summary graph n = 7, *** p<0.001, one-way ANOVA with Bonferroni post test are shown.
(TIF)

Figure S4 **Lack of apoptosis in CD3^+^ purified T-cells is not due to altered kinetics of apoptosis induction.** Peripheral blood mononuclear cells (PBMC) and highly purified T -cells (CD3 enriched) were either mock-infected (MI) for 2–10 h A) or 10–16 h B) or exposed to serotype 2 *Streptococcus pneumoniae* (D39) (MOI = 50) for 2–10 h C) or 10–16 h D). Apoptosis was recorded in CD3^+^ T-cells measuring hypodiploid DNA content (Sub G0/1). n = 4, ** p<0.01, statistical analysis by two -way ANOVA.
(TIFF)

Figure S5 **Neutrophils do not alter the ability of monocytes to induce T-cell death.** Peripheral blood mononuclear cells were mock infected (MOI = 0) or challenged with *Streptococcus pneumoniae* serotype 2 (D39) (MOI = 10) for 6 h in the absence of neutrophils (Control), in the presence of neutrophils (with PMN) or the presence of apoptotic neutrophils (with apoptotic PMN). CD3^+^ T-cell apoptosis was measured as the percentage of cells with hypodiploid DNA n = 4, * p<0.05, *** p<0.001, ns = not significant, two-way ANOVA with Bonferroni post-test.
(TIF)

Figure S6 **The percentage of monocyte sub-populations in monocytes isolated using different immunoselection protocols.** A) Representative dot plots showing isotype (left panel) and CD16 and CD14 positive staining of human peripheral blood monocytes isolated by magnetic immunoselection using a pan monocyte isolation kit (middle panel) and a ‘classical’ monocyte isolation kit (right panel); and B) mean and standard error of the mean percentage cells in each sub-population of monocytes. Monocyte subsets were divided into ‘classical’ (CD14^++^ CD16^−^), ‘intermediate’ (CD14^++^ CD16^+^), and ‘non-classical’ (CD14^lo^ CD16^+^), n = 4.
(TIF)

Figure S7 **CD3^+^ purified T-cells in PBMC cocultures die by a caspase-1 independent death mechanism that requires live bacteria.** A) Western blot probed for active caspase 1 and actin from both peripheral blood mononuclear cells (PBMC) and purified CD3^+^ T-cells (CD3 enriched) 16 h following mock-infection (D39−) or challenge with serotype 2 pneumococci (D39+) at MOI = 10 in the presence of isotype control (Isotype+) or ZB4 neutralizing anti-Fas antibody (Anti-Fas+). The positive control (+ve) is THP-1 cells infected with *E.coli* a known stimulus for pyroptosis [15]. B) Cell death was measured using flow cytometry to detect Annexin V^+^ events in peripheral blood lymphocytes (PBL) gated by forward (FSC) and side scatter (SSC) 4 h after mock-infection (multiplicity of infection (MOI) = 0) or challenge of peripheral blood mononuclear cells (PBMC) with live or heat killed D39 *Streptococcus pneumoniae* (MOI = 10), n = 5. C) Red blood cells were incubated with samples from serotype 2 *S. pneumoniae* (D39), its Æ6 mutant expressing non-cytolytic pneumolysin, a pneumolysin deficient D39 mutant (PLY−), 1 µg/ml exogenous pneumolysin (ply), PBS as a negative control or water as a positive control. n = 3, * p<0.05, ** p<0.01 statistical analysis by ANOVA.
(TIFF)

Figure S8 **Granzyme B does not induce lymphocyte apoptosis.** Peripheral blood mononuclear cells (PBMCs) were mock-infected (D39−) or infected with serotype 2 *Streptococcus pneumoniae* (D39+) at a multiplicity of infection of 50 for 6 h in the presence of the vehicle control (DMSO) or the granzyme B inhibitor (GBI) at 5 µM. Cells were harvested and the percentage of cells with sub G0/1 DNA (Sub G0/1) estimated by PI staining, n = 4.
(TIF)

Figure S9 **Characterization of hypodiploid DNA.** Peripheral blood mononuclear cells (PBMCs) were mock-infected (MI) or infected with serotype 2 *Streptococcus pneumoniae* (D39), or serotype 1 strains with (INV104B) or without (NCTC7465) endonuclease A activity, at multiplicity of infection (MOI) = 50. Some NCTC7465 cultures were also incubated with DNase (+DNase). Six hours post-infection T-cells were identified by labeling with FITC conjugated anti-CD3. The percentage of sub G0/1 CD3^+^ T-cells was identified using propidium iodide (PI) staining. A) Representative dot plots showing gating of the PBMC population using FSC vs SSC (left panel), isotype staining (middle panel) and staining of CD3^+^ T-cells (right panel) with the percentage of cells in the indicated region shown in the upper right hand corner. B) Representative histograms showing the percentage sub G0/1 CD3^+^ T-cells including (top gate) and excluding (bottom gate) events in the far left sub G0/1 peak. C) Graph showing percentage of sub G0/1 CD3^+^ T-cells excluding events in the far left sub G0/1 peak (n = 3) following each treatment.
(TIFF)
