# Supplementary figures and images for: Monocytes Regulate the Mechanism of T-cell Death by Inducing Fas-Mediated Apoptosis during Bacterial Infection
Source: PLoS Pathog. 2012 Jul 19;8(7):e1002814. doi: 10.1371/journal.ppat.1002814 (PMC3400568; doi:10.1371/journal.ppat.1002814)

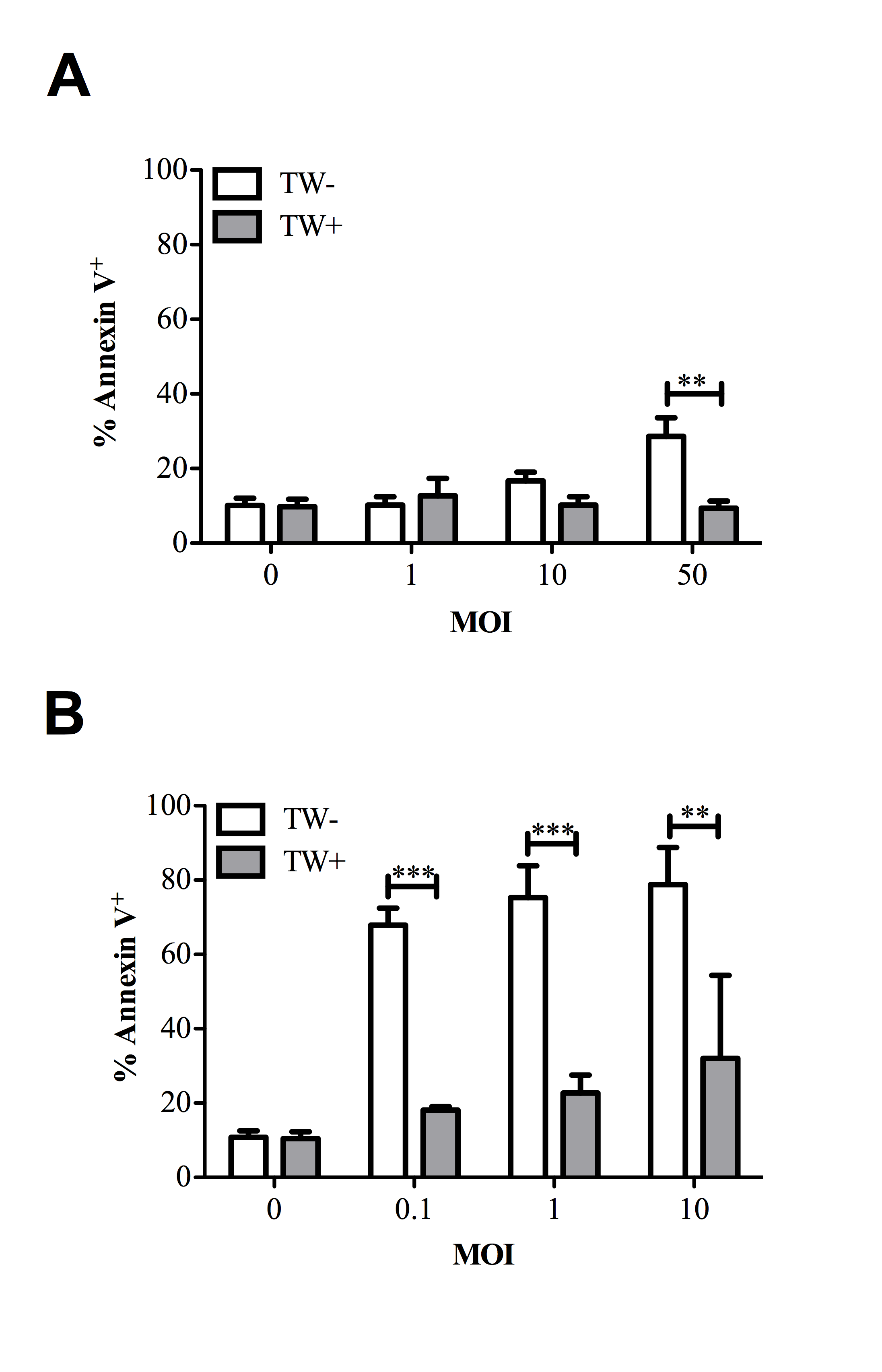

Supplement: Figure S1 — Cell contact is required for lymphocyte death. Peripheral blood lymphocytes (PBL) were isolated by plastic adherence and mock infected (MOI = 0) or challenged with Streptococcus pneumoniae serotype 2 (D39). (MOI = 0.1–50) in the presence (TW+) or absence (TW−) of semi -permeable transwell membranes. A) PBL were challenged for 6 h, MOI = 0, 1, 10 or 50 or B) 24 hours, MOI = 0, 0.1, 1 or 10. Cell death was recorded by flow cytometry in lymphocytes gated on forward light scatter and side light scatter characteristics and stained with Annexin V and TOPRO-3. All Annexin V positive (Annexin V+) cells were treated as dead, n = 3, *** p<0.001, two-way ANOVA with Bonferroni post-test. (TIFF) [file ppat.1002814.s001.tiff]

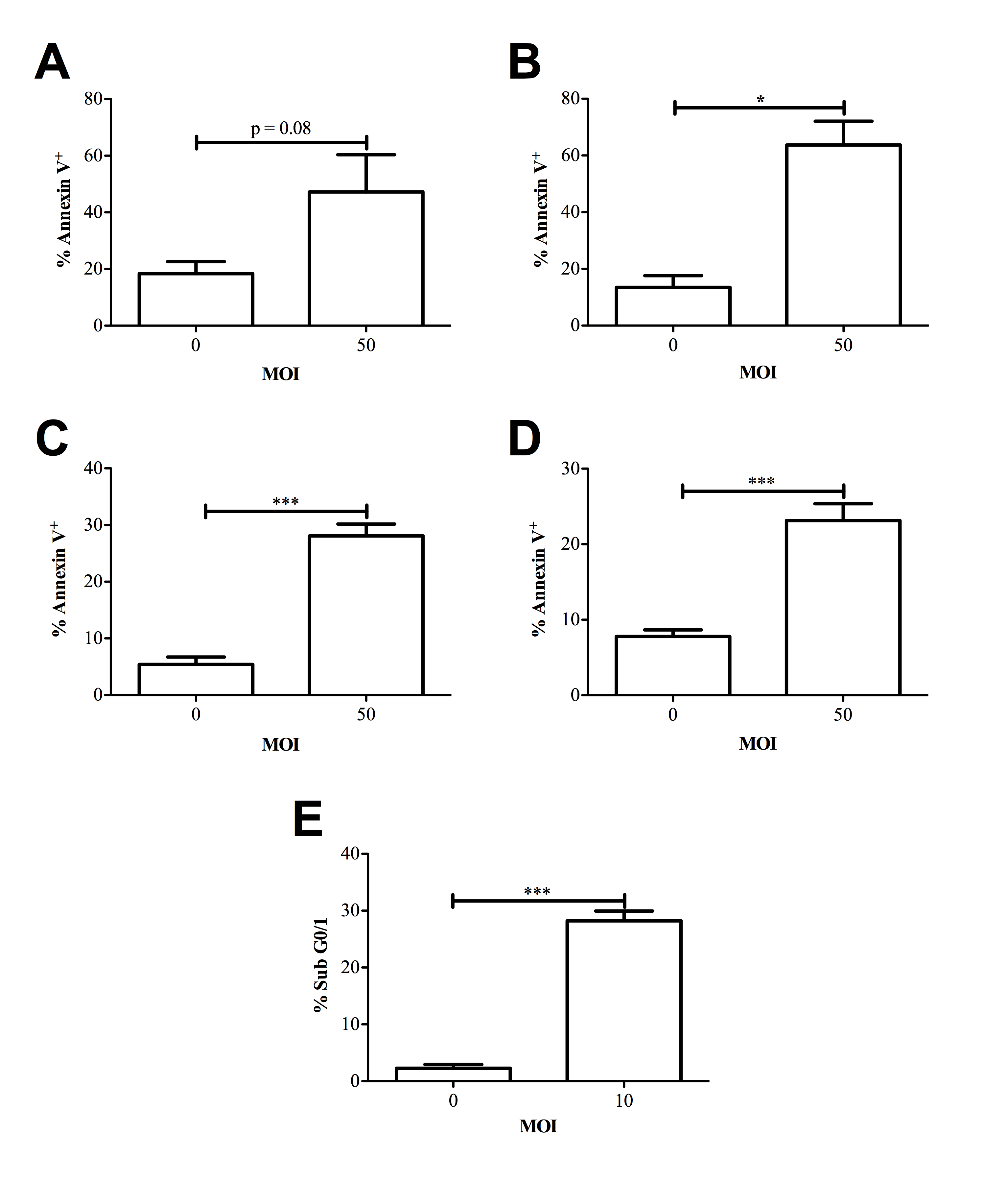

Supplement: Figure S2 — Sub-sets of peripheral blood mononuclear cells are susceptible to cell death following pneumococcal challenge. The percentage Annexin V+ cells in subsets of peripheral blood mononuclear cells was estimated after mock-infection (MOI 0) or challenge with Streptococcus pneumoniae (MOI = 10 or 50 as indicated) and staining with anti -CD19, anti-CD14, anti-CD3 plus anti-CD4 or anti -CD3 plus anti -CD161. A) CD19+ B-cells after 16 h challenge, and B) CD14+ monocytes after 6 h challenge, C) CD3+/CD4+ T-cells and D) CD3+/CD4+ T-cells after 6 h challenge (all MOI = 50) and E) the percentage CD3+/CD161+ T-cells with hypodiploid DNA (Sub G0/1) after 16 h challenge (MOI = 10). n = 4, * p<0.05, *** p<0.001 statistical analysis by t -test. (TIFF) [file ppat.1002814.s002.tiff]

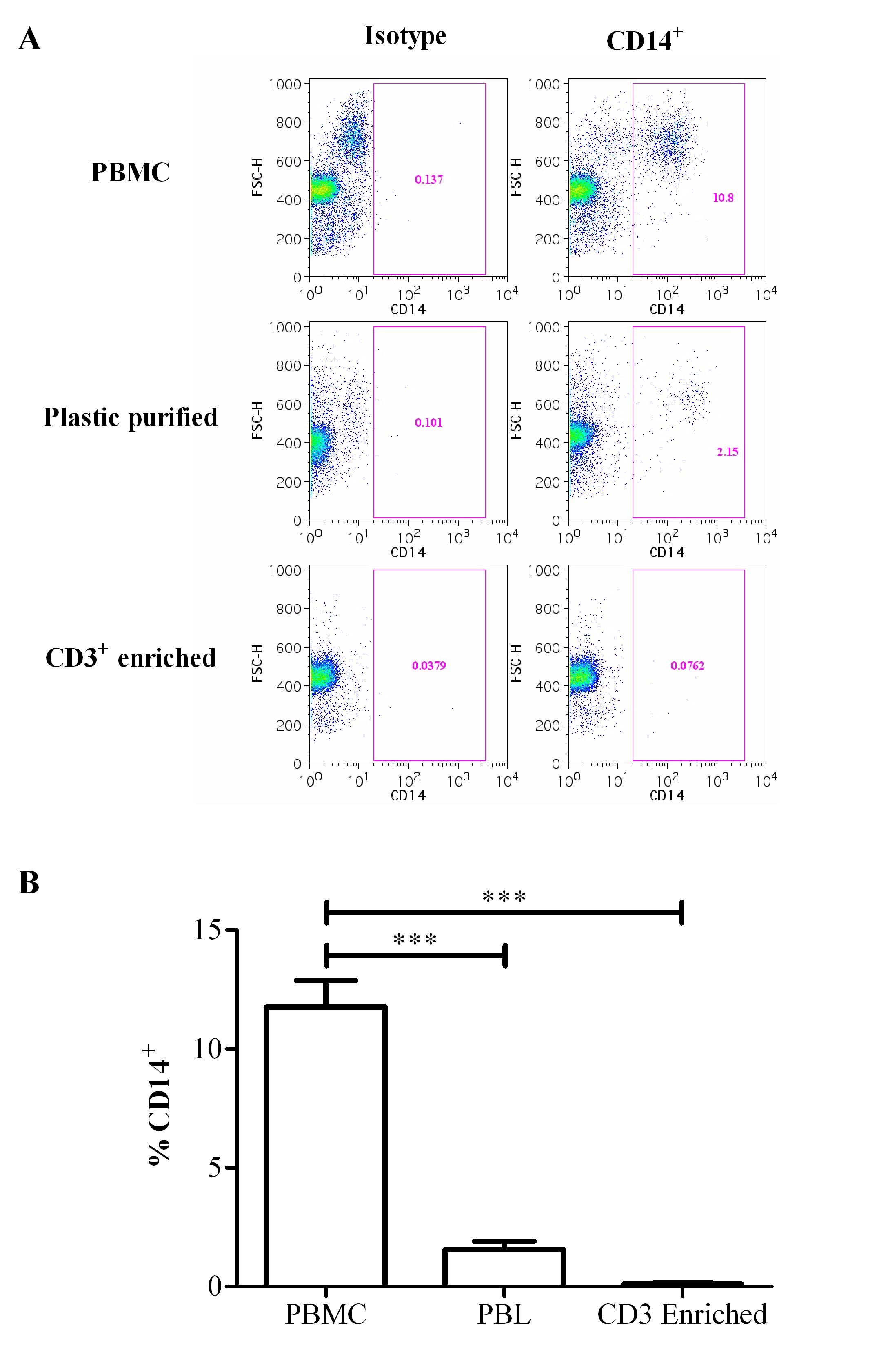

Supplement: Figure S3 — CD14+ contaminating monocytes are effectively removed by CD3+ T-lymphocyte magnetic immunoselection. Levels of CD14+ monocytes were measured in peripheral blood mononuclear cell (PBMC) populations subjected to various purification methods. PBMC, plastic purified lymphocytes (Plastic purified) and PBMC highly purified by magnetic immunoselection to yield CD3+ T-lymphocytes (CD3 enriched) at high purity were stained with mouse anti-human CD14 or isotype, and analysed by flow cytometry. A) Representative dot plots and B) a summary graph n = 7, *** p<0.001, one-way ANOVA with Bonferroni post test are shown. (TIF) [file ppat.1002814.s003.tiff]

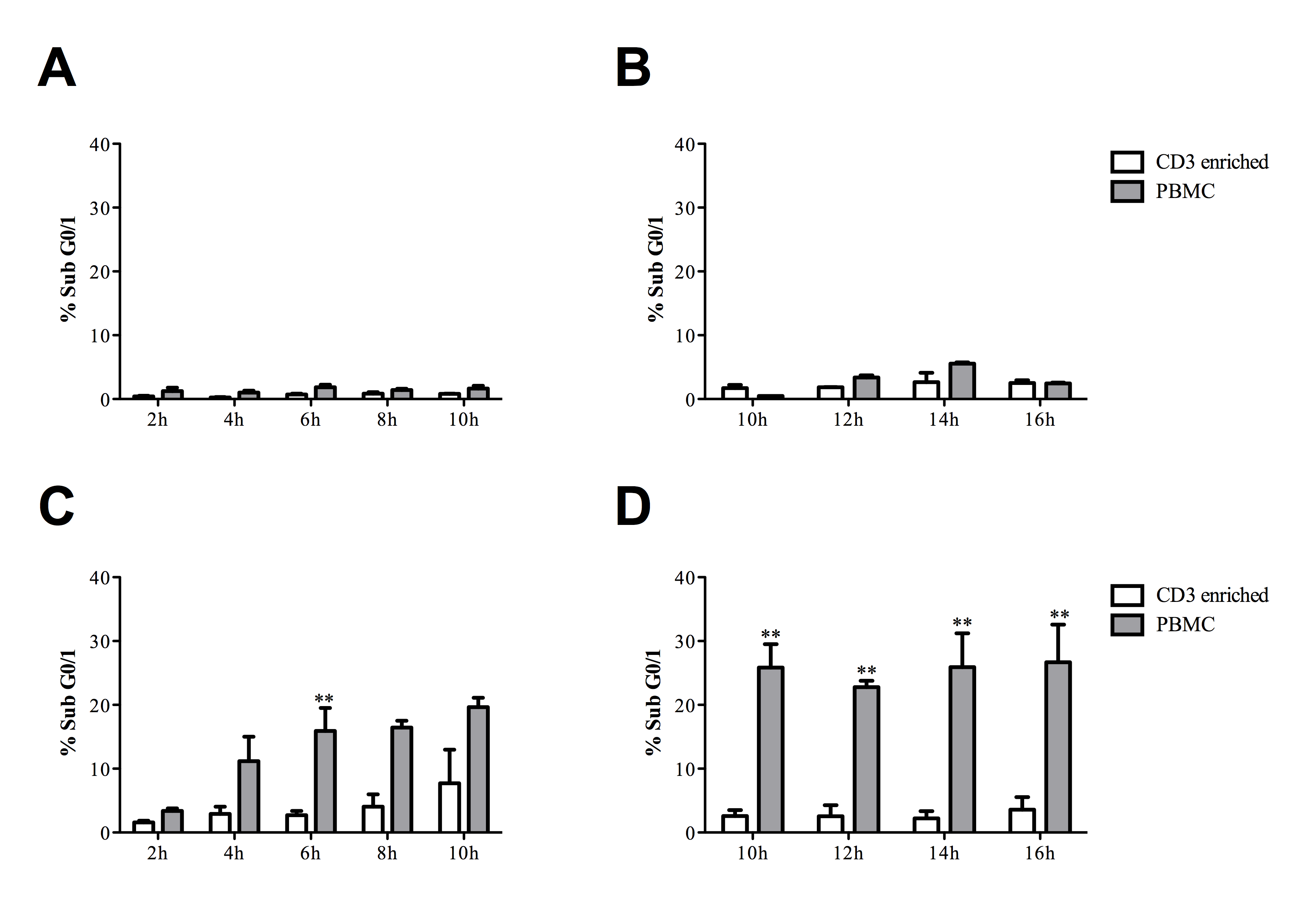

Supplement: Figure S4 — Lack of apoptosis in CD3+ purified T-cells is not due to altered kinetics of apoptosis induction. Peripheral blood mononuclear cells (PBMC) and highly purified T -cells (CD3 enriched) were either mock-infected (MI) for 2–10 h A) or 10–16 h B) or exposed to serotype 2 Streptococcus pneumoniae (D39) (MOI = 50) for 2–10 h C) or 10–16 h D). Apoptosis was recorded in CD3+ T-cells measuring hypodiploid DNA content (Sub G0/1). n = 4, ** p<0.01, statistical analysis by two -way ANOVA. (TIFF) [file ppat.1002814.s004.tiff]

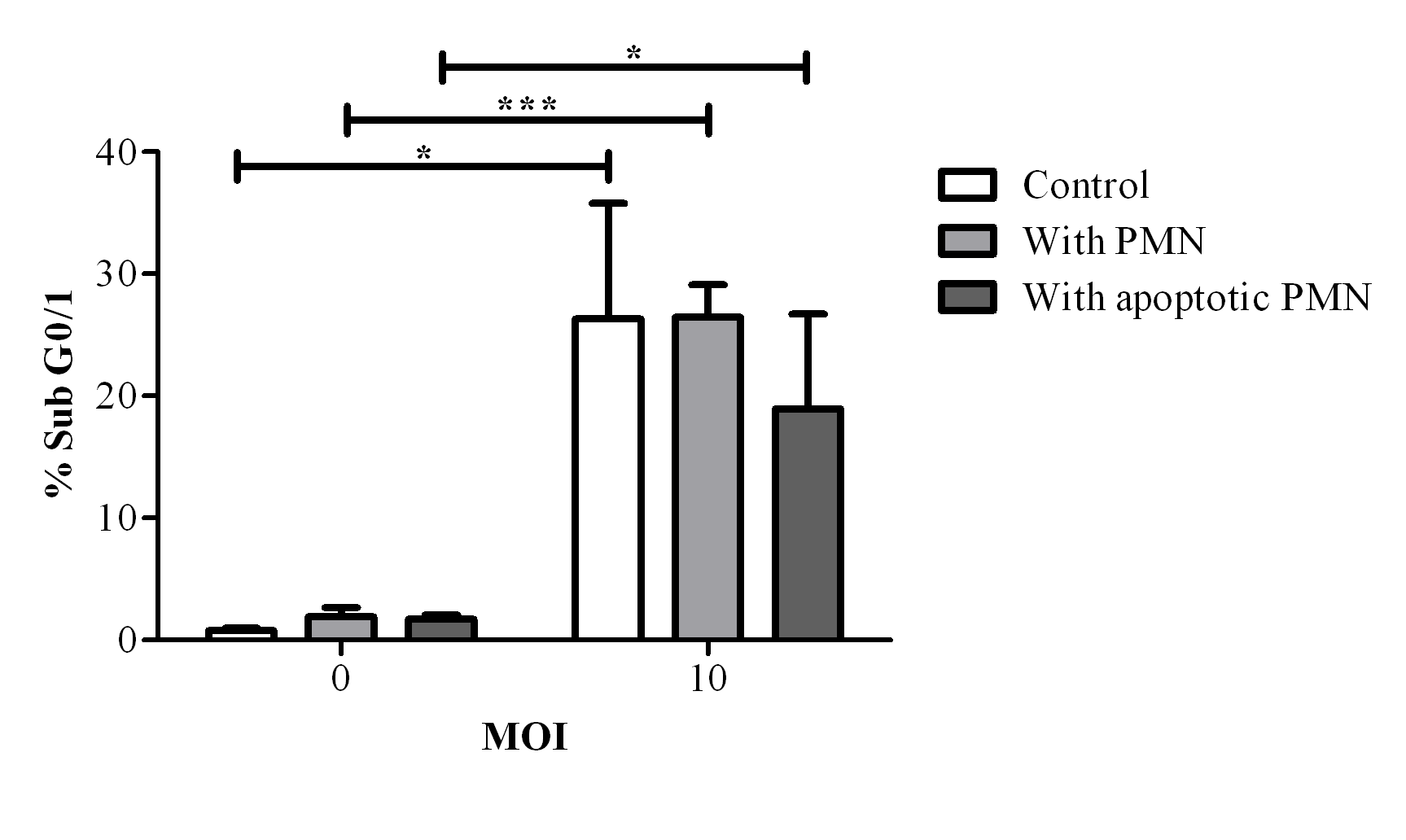

Supplement: Figure S5 — Neutrophils do not alter the ability of monocytes to induce T-cell death. Peripheral blood mononuclear cells were mock infected (MOI = 0) or challenged with Streptococcus pneumoniae serotype 2 (D39) (MOI = 10) for 6 h in the absence of neutrophils (Control), in the presence of neutrophils (with PMN) or the presence of apoptotic neutrophils (with apoptotic PMN). CD3+ T-cell apoptosis was measured as the percentage of cells with hypodiploid DNA n = 4, * p<0.05, *** p<0.001, ns = not significant, two-way ANOVA with Bonferroni post-test. (TIF) [file ppat.1002814.s005.tiff]

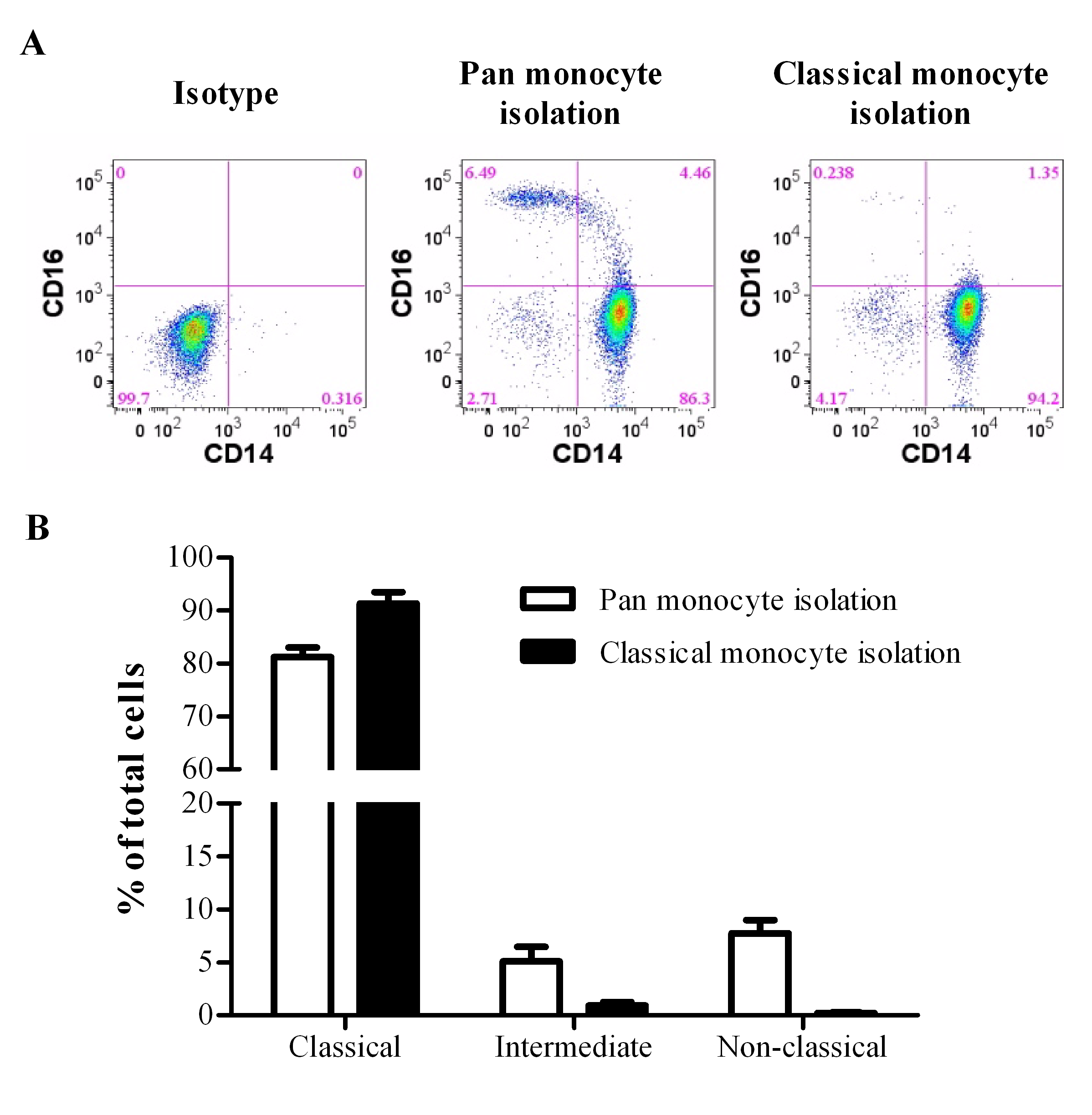

Supplement: Figure S6 — The percentage of monocyte sub-populations in monocytes isolated using different immunoselection protocols. A) Representative dot plots showing isotype (left panel) and CD16 and CD14 positive staining of human peripheral blood monocytes isolated by magnetic immunoselection using a pan monocyte isolation kit (middle panel) and a ‘classical’ monocyte isolation kit (right panel); and B) mean and standard error of the mean percentage cells in each sub-population of monocytes. Monocyte subsets were divided into ‘classical’ (CD14++ CD16−), ‘intermediate’ (CD14++ CD16+), and ‘non-classical’ (CD14lo CD16+), n = 4. (TIF) [file ppat.1002814.s006.tiff]

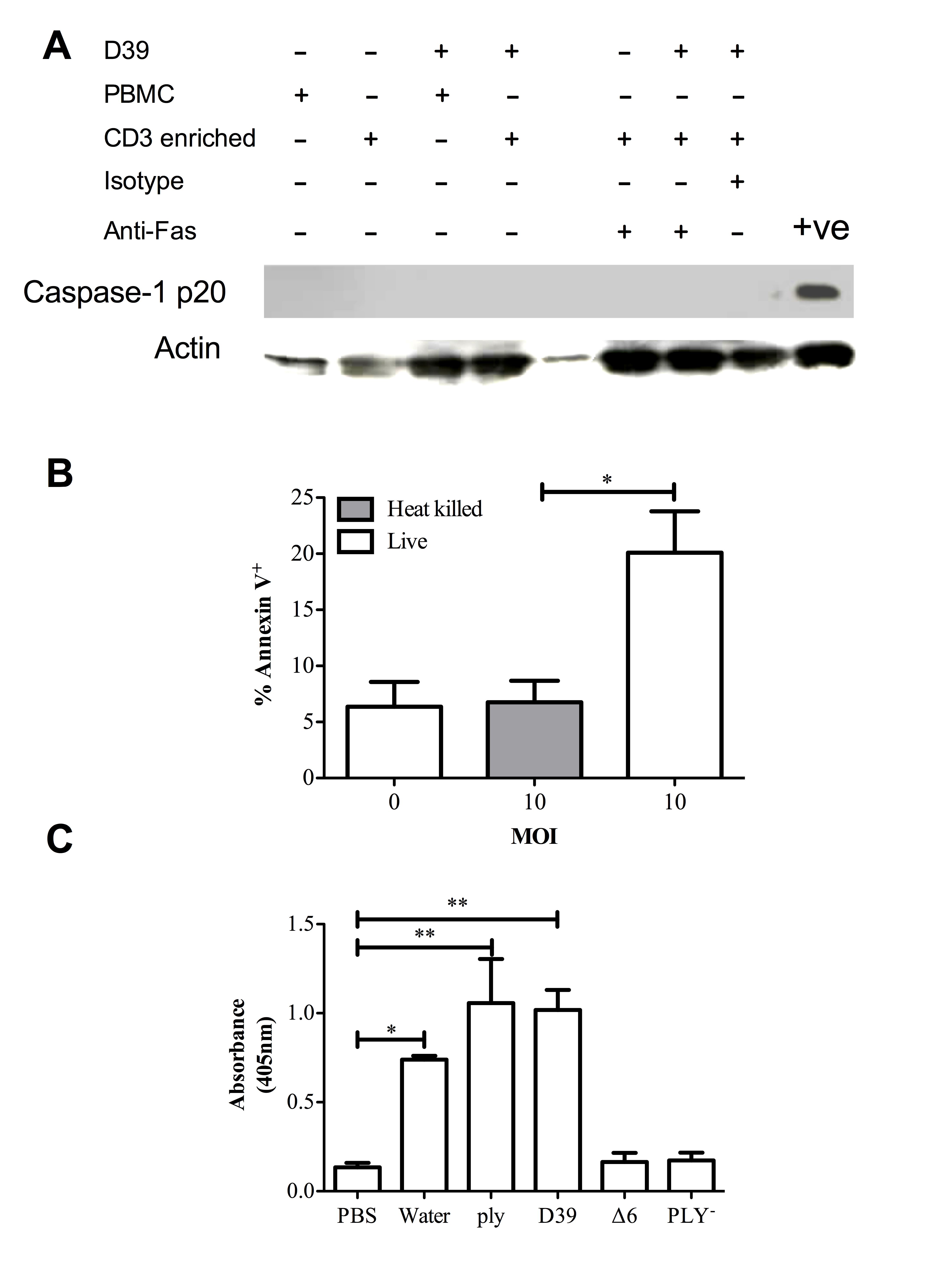

Supplement: Figure S7 — CD3+ purified T-cells in PBMC cocultures die by a caspase-1 independent death mechanism that requires live bacteria. A) Western blot probed for active caspase 1 and actin from both peripheral blood mononuclear cells (PBMC) and purified CD3+ T-cells (CD3 enriched) 16 h following mock-infection (D39−) or challenge with serotype 2 pneumococci (D39+) at MOI = 10 in the presence of isotype control (Isotype+) or ZB4 neutralizing anti-Fas antibody (Anti-Fas+). The positive control (+ve) is THP-1 cells infected with E.coli a known stimulus for pyroptosis [15]. B) Cell death was measured using flow cytometry to detect Annexin V+ events in peripheral blood lymphocytes (PBL) gated by forward (FSC) and side scatter (SSC) 4 h after mock-infection (multiplicity of infection (MOI) = 0) or challenge of peripheral blood mononuclear cells (PBMC) with live or heat killed D39 Streptococcus pneumoniae (MOI = 10), n = 5. C) Red blood cells were incubated with samples from serotype 2 S. pneumoniae (D39), its Æ6 mutant expressing non-cytolytic pneumolysin, a pneumolysin deficient D39 mutant (PLY−), 1 µg/ml exogenous pneumolysin (ply), PBS as a negative control or water as a positive control. n = 3, * p<0.05, ** p<0.01 statistical analysis by ANOVA. (TIFF) [file ppat.1002814.s007.tiff]

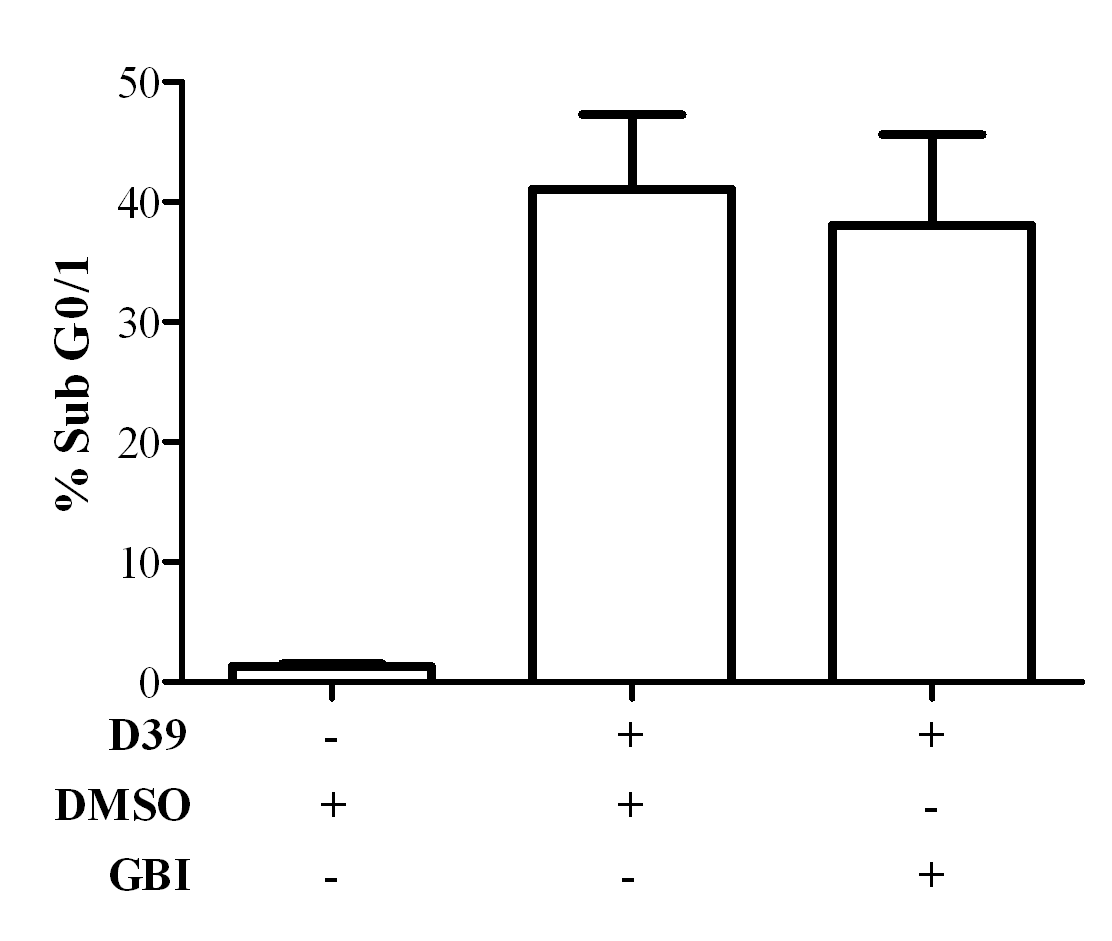

Supplement: Figure S8 — Granzyme B does not induce lymphocyte apoptosis. Peripheral blood mononuclear cells (PBMCs) were mock-infected (D39−) or infected with serotype 2 Streptococcus pneumoniae (D39+) at a multiplicity of infection of 50 for 6 h in the presence of the vehicle control (DMSO) or the granzyme B inhibitor (GBI) at 5 µM. Cells were harvested and the percentage of cells with sub G0/1 DNA (Sub G0/1) estimated by PI staining, n = 4. (TIF) [file ppat.1002814.s008.tiff]

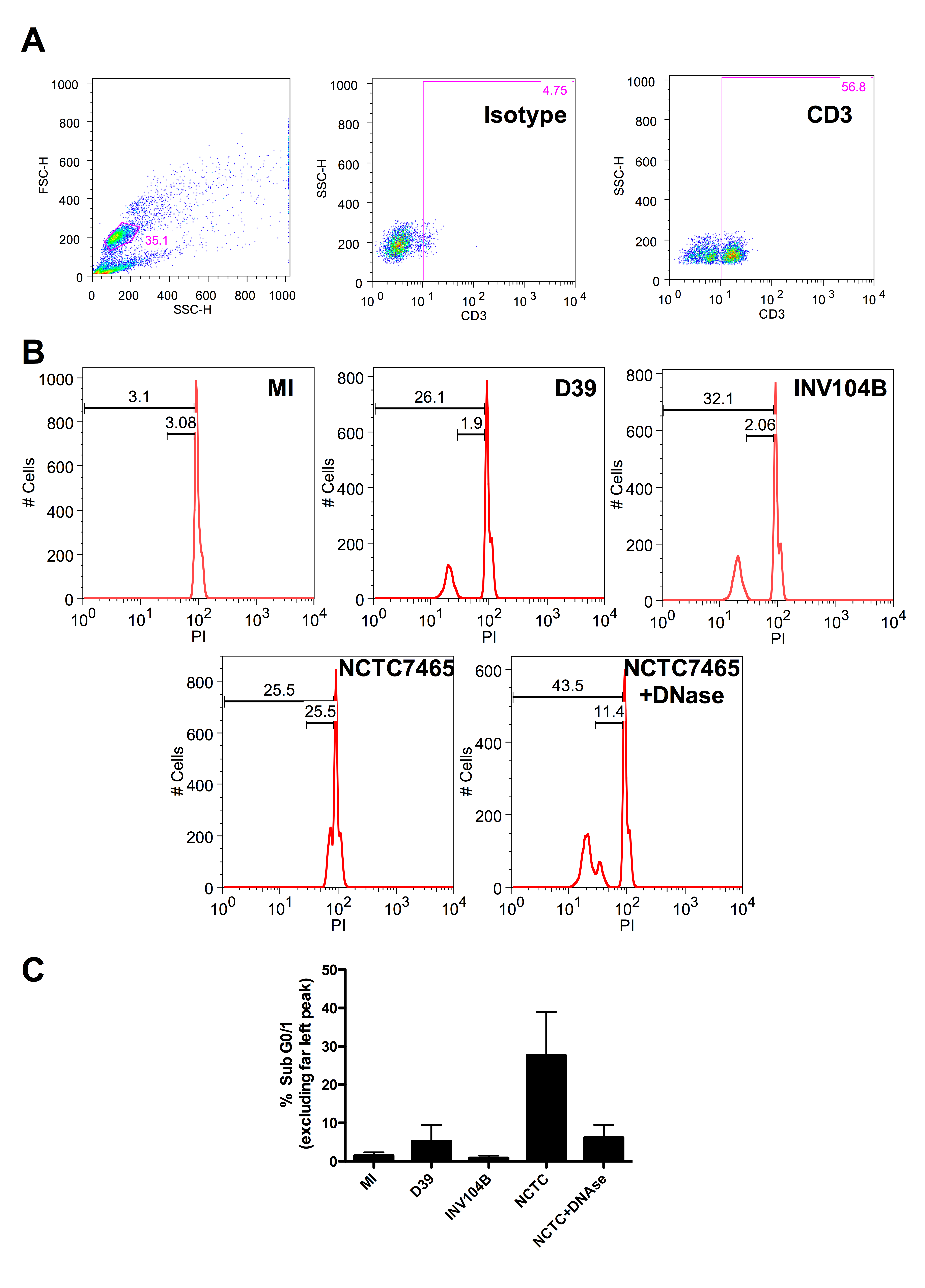

Supplement: Figure S9 — Characterization of hypodiploid DNA. Peripheral blood mononuclear cells (PBMCs) were mock-infected (MI) or infected with serotype 2 Streptococcus pneumoniae (D39), or serotype 1 strains with (INV104B) or without (NCTC7465) endonuclease A activity, at multiplicity of infection (MOI) = 50. Some NCTC7465 cultures were also incubated with DNase (+DNase). Six hours post-infection T-cells were identified by labeling with FITC conjugated anti-CD3. The percentage of sub G0/1 CD3+ T-cells was identified using propidium iodide (PI) staining. A) Representative dot plots showing gating of the PBMC population using FSC vs SSC (left panel), isotype staining (middle panel) and staining of CD3+ T-cells (right panel) with the percentage of cells in the indicated region shown in the upper right hand corner. B) Representative histograms showing the percentage sub G0/1 CD3+ T-cells including (top gate) and excluding (bottom gate) events in the far left sub G0/1 peak. C) Graph showing percentage of sub G0/1 CD3+ T-cells excluding events in the far left sub G0/1 peak (n = 3) following each treatment. (TIFF) [file ppat.1002814.s009.tiff]
